# HAM-5 Functions As a MAP Kinase Scaffold during Cell Fusion in *Neurospora crassa*

**DOI:** 10.1371/journal.pgen.1004783

**Published:** 2014-11-20

**Authors:** Wilfried Jonkers, Abigail C. Leeder, Charles Ansong, Yuexi Wang, Feng Yang, Trevor L. Starr, David G. Camp, Richard D. Smith, N. Louise Glass

**Affiliations:** 1 Department of Plant and Microbial Biology, University of California, Berkeley, Berkeley, California, United States of America; 2 Pacific Northwest National Laboratory, Richland, Washington, United States of America; 3 Energy Biosciences Institute, University of California, Berkeley, Berkeley, California, United States of America; Duke University Medical Center, United States of America

## Abstract

Cell fusion in genetically identical *Neurospora crassa* germlings and in hyphae is a highly regulated process involving the activation of a conserved MAP kinase cascade that includes NRC-1, MEK-2 and MAK-2. During chemotrophic growth in germlings, the MAP kinase cascade members localize to conidial anastomosis tube (CAT) tips every ∼8 minutes, perfectly out of phase with another protein that is recruited to the tip: SOFT, a recently identified scaffold for the MAK-1 MAP kinase pathway in *Sordaria macrospor*a. How the MAK-2 oscillation process is initiated, maintained and what proteins regulate the MAP kinase cascade is currently unclear. A global phosphoproteomics approach using an allele of *mak-2* (*mak-2^Q100G^*) that can be specifically inhibited by the ATP analog 1NM-PP1 was utilized to identify MAK-2 kinase targets in germlings that were potentially involved in this process. One such putative target was HAM-5, a protein of unknown biochemical function. Previously, Δ*ham-5* mutants were shown to be deficient for hyphal fusion. Here we show that HAM-5-GFP co-localized with NRC-1, MEK-2 and MAK-2 and oscillated with identical dynamics from the cytoplasm to CAT tips during chemotropic interactions. In the Δ*mak-2* strain, HAM-5-GFP localized to punctate complexes that did not oscillate, but still localized to the germling tip, suggesting that MAK-2 activity influences HAM-5 function/localization. However, MAK-2-GFP showed cytoplasmic and nuclear localization in a Δ*ham-5* strain and did not localize to puncta. Via co-immunoprecipitation experiments, HAM-5 was shown to physically interact with NRC-1, MEK-2 and MAK-2, suggesting that it functions as a scaffold/transport hub for the MAP kinase cascade members for oscillation and chemotropic interactions during germling and hyphal fusion in *N. crassa*. The identification of HAM-5 as a scaffold-like protein will help to link the activation of MAK-2 cascade to upstream factors and proteins involved in this intriguing process of fungal communication.

## Author Summary

Cell fusion between genetically identical cells of the fungus *Neurospora crassa* occurs when germinating asexual cells (conidia) sense each other's proximity and redirect their growth. Chemotropic growth is dependent upon the assembly of a MAPK cascade (NRC-1/MEK-2/MAK-2) at the cell cortex (conidial anastomosis tubes; CATs), followed by disassembly over an ∼8 min cycle. A second protein required for fusion, SO, also assembles and disassembles at CAT tips during chemotropic growth, but with perfectly opposite dynamics to the MAK-2 complex. This process of germling chemotropism, oscillation and cell fusion is regulated by many genes and is poorly understood. Via a phosphoproteomics approach, we identify HAM-5, which functions as a scaffold for the MAK-2 signal transduction complex. HAM-5 is required for assembly/disassembly and oscillation of the MAK-2 complex during chemotropic growth. Our data supports a model whereby regulated modification of HAM-5 controls the disassembly of the MAK-2 MAPK complex and is essential for modulating the tempo of oscillation during chemotropic interactions.

## Introduction

Fusion between genetically identical cells occurs in many different organisms and plays pivotal roles in different developmental processes, such as myoblast fusion during muscle formation, macrophage fusion involved in tissue remodeling and fusion of trophoblasts during placental development [1], [2]. Cell fusion is also important for the formation of the interconnected mycelial network that is the hallmark of filamentous fungal growth [3], [4], [5]. In addition to hyphal fusion, fusion can also occur between genetically identical germinating asexual spores (conidia) of filamentous fungi [6], [7], [8]. Both hyphal and germling fusion are integral to the formation of an interconnected hyphal network and impart fitness benefits, as well as mediating genetic mixing and the sharing of resources [3], [4], [9], [10], [11], [12], [13].

In the filamentous ascomycete fungus, *Neurospora crassa*, germinated conidia (germlings) in close proximity fuse via specialized conidial anastomosis tubes (CATs) that form at germ tube tips or between conidia [14]. In a fungal colony, hyphal fusion is observed in the central parts of the colony, in contrast to the peripheral parts where hyphae avoid each other. A large number of genes have been identified in *N. crassa* that are important for the process of sensing, chemotropic interactions and CAT fusion and have contributed to an understanding of this complex developmental system in filamentous ascomycete fungi [7], [15], [16], [17]. An essential part of chemotropic interactions in *N. crassa* is the oscillatory recruitment of three kinases of a MAPK cascade (NRC-1, MEK-2 and MAK-2) and of a protein of unknown function, SOFT (SO), to CAT tips [18], [19]. In strains carrying loss-of-function mutations in these genes, oscillatory recruitment of NRC-1/MEK-2/MAK-2 or SO, chemotropic interactions and fusion do not occur [8], [18], [19], [20]. It was proposed that the alternating oscillation of MAK-2 and SO to CAT tips may function to establish two distinct physiological states in interacting germlings to enable chemotropism to persist, avoid self-stimulation and assure a rapid and efficient cell fusion [19], [21]. Recently, it has been shown that an ortholog of SOFT in the related filamentous ascomycete species, *Sordaria macrospora*, PRO40, is a scaffold protein for the cell wall integrity MAP kinase pathway (MAK-1) [22]; Δ*mak-1* mutants in both *N. crassa* and *S. macrospora* are fusion mutants [22], [23].

Previously, it was shown that kinase activity of MAK-2 is required to maintain oscillatory recruitment of both MAK-2 and SO; addition of ATP-analog 1NM-PP1 to a mutant containing an inhibitable MAK-2 protein encoded by a *mak-2^Q100G^* allele, disrupted the oscillation of both MAK-2 and SO in communicating germlings and stalled chemotropic interactions and the fusion process [19]. The use of *mak-2^Q100G^* strain also contributed to the identification of downstream genes whose expression levels depend on functional MAK-2 [24]. However, MAK-2 kinase targets involved in the oscillation process have not been identified and the characterization of such potential targets could help unravel molecular mechanisms associated with this highly regulated and complex process.

In recent years, highly sensitive liquid chromatography-mass spectrometry (LC-MS) based quantitative phosphoproteomic techniques have contributed to our understanding of kinase pathway function in eukaryotic cells [25], [26], [27], [28]. To identify MAK-2 kinase targets in *N. crassa*, we took a global approach by identifying phosphopeptides in *mak-2^Q100G^* germlings, treated or not with 1NM-PP1. From the phosphoproteomic screen, a number of candidate MAK-2 target proteins were identified, one of which encodes a protein previously identified as being essential for germling/hyphal fusion, HAM-5 [23], [29]. We show that HAM-5 oscillates during chemotropic interactions with components of the MAK-2 pathway, physically interacts with NRC-1, MEK-2 and MAK-2 and was required for localization of MAK-2 and MEK-2 to puncta. Our data supports the hypothesis that HAM-5 functions as a scaffold protein by binding to and co-localizing to CAT tips with all three kinases in the MAK-2 cascade during chemotropic growth in germlings as well as in hyphae undergoing fusion events. These studies shed new light on the mechanisms of oscillation during communication and chemotropic interactions between genetically identical cells and which may be important for function of this conserved MAPK pathway in other filamentous ascomycete fungi.

## Results

### Identification of MAK-2 targets in Δ*mak-2^Q100G^* germlings using a phosphoproteomic approach

To better understand the role of MAK-2 during chemotropic interactions, we set out to identify putative kinase targets using a global quantitative phosphoproteomics approach using a strain carrying an inhibitable *mak-2^Q100G^* allele. Altering a specific amino acid in the ATP binding site (glutamine for glycine) renders MAK-2 sensitive to inhibition to the ATP analogue 1NM-PP1, but does not affect MAK-2 kinase activity in the absence of inhibitor [19]. In the absence of the inhibitor 1NM-PP1, strains and germlings containing the *mak-2^Q100G^* allele (*his3::mak-2^Q100G^*; *Δmak-2*) showed wild-type growth and fused normally, while in the presence of inhibitor, the *mak-2^Q100G^* strain showed a mutant *mak-2* phenotype; chemotropic interactions and fusion were not observed, consistent with inactivation of MAK-2 kinase activity [19]. For identifying MAK-2-dependent phosphorylation events, 4.5-hr old *mak-2^Q100G^* germlings were treated with DMSO (two samples and two biological replicates) or 1NM-PP1 (dissolved in DMSO; two samples and two biological replicates) for 10 min. Proteins were extracted, digested, and enriched phosphopeptides identified and quantified using isobaric peptide tags for relative and absolute quantification (iTRAQ)-based LC-MS/MS analyses. Although the same peptides across experimental conditions are labeled with different iTRAQ reagents and indistinguishable by mass, different masses will be generated in the tandem MS by releasing the reporter ions for the 4-plex iTRAQ method (Figure S1). We performed the full experiment twice, resulting in a total of eight samples, which were analyzed for phosphopeptide identity and abundance.

**Figure 1:**
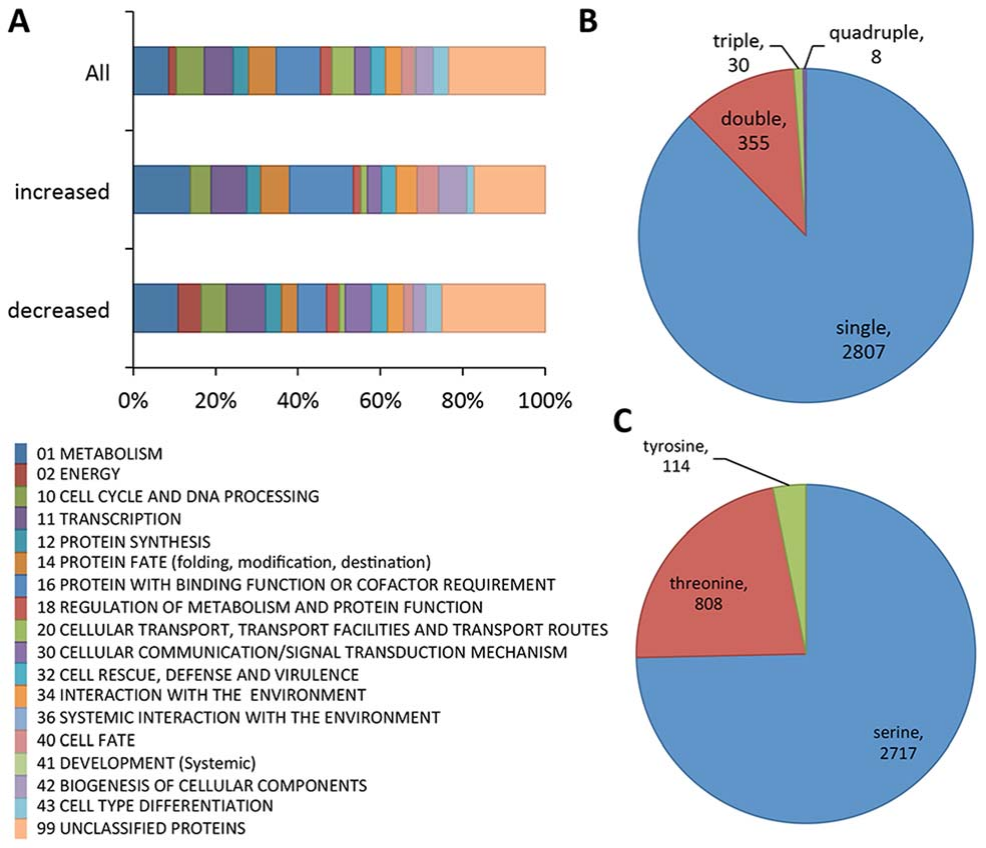
Summary of phosphoproteomics results conducted on Δ*mak-2^Q100G^* mutant. (A) Overview of FunCat categories of proteins harboring the identified phosphopeptides. The upper bar shows the categories of all the proteins with identified phosphopeptides (3200 phosphopeptides, 1164 proteins), the middle bar shows functional categories of proteins with phosphopeptides that showed higher abundance after inhibition (33 phosphopeptides, 27 proteins) and the lower bar shows FunCat analysis of the proteins with phosphopeptides that showed lower abundance in *mak-2^Q100G^* germlings after treatment with 1NM-PP1 (96 phosphopeptides, 67 proteins). (B) Pie chart showing the relative percentages and absolute numbers of single, double, triple and quadruple phosphosites per peptide. (C) Pie chart showing the relative percentages and absolute numbers of phosphorylated serine, threonine and tyrosine in all peptides found.

From these experiments a total of 3200 unique phosphopeptides were identified. These 3200 unique phosphopeptides originated from 1164 proteins (Dataset S1). A small percentage of the identified peptides have multiple phosphorylation sites (12%, Figure 1B), as compared to peptides where only a single phosphorylation site was identified (88%, Figure 1B). Phosphorylation sites were predominantly identified on serine residues (75%), and to a lesser extent on threonine (22%) and tyrosine (3%) residues (Figure 1C). FunCat analysis [30] showed the set of identified phosphorylated proteins in germlings originated from a wide spectrum of functional categories (Figure 1A), including, not unexpectedly, proteins involved in metabolism, energy, cell cycle and DNA processing, protein synthesis and transcription. However, proteins within the functional categories of cellular communication/signal transduction, cell defense, and interaction with the environment were also identified, suggesting germlings are poised to respond to variations in their environment (Dataset S1).

Of the 3200 phosphopeptides, 96 unique phosphopeptides from 67 proteins were>1.5 times less abundant (p<0.05) in at least one replica experiment in the 1NM-PP1 treated *mak-2^Q100G^* germlings relative to the DMSO-treated control cells (Table S1). Functional category analyses [30] of this set of proteins showed enrichment for genes involved in metabolism, energy, cell cycle and DNA processing, transcription, protein fate, regulation of metabolism and protein function, cellular communication/signal transduction, interaction with the environment, cell fate and cell type differentiation (p<0.01) (Figure 1A; Dataset S1). Three proteins in this group had previously been shown to be required for hyphal fusion, including HAM-9, HAM-11 and MAK-1 [23], [24], [31], [32]. In addition to the MAP kinase involved in the osmotic response signaling (OS2) [32], CUT-1, which is implicated in the osmotic stress response [33], [34] as well as the transcription factor that is a target of the OS-2 pathway (ASL-1/ATF-1, NCU01345) [35] were identified (Table 1; Table S1). Of these 3200 unique phosphopeptides, 33 phosphopeptides (from 27 proteins) were identified with an increased abundance of at least 1.5 fold (p<0.05) in the 1NM-PP1 treated germlings in at least one replica experiment. Functional category analyses [30] of this set of 27 proteins showed over-representation for genes/proteins involved in phosphate metabolism (perhaps as a response to 1NM-PP1 exposure), protein synthesis, protein fate and protein with binding function or cofactor requirements (Dataset S1).

**Table 1:** Phosphopeptides with predicted MAPK site that showed decreased abundance in 1NM-PP1-treated *mak-2^Q100G^* cells.

NCU #	Phosphorylated peptide^1^	Annotation	locus	Fusion
NCU00521^2^	SPVS*PTAGEFTFAPRQS*LDSAR	hypothetical protein		yes
NCU01345	R.SGPLS*PAMLSGPTTSDYFGDHIR	*ascospore lethal-1*	*asl-1*	yes
NCU01728	SLNDLDGGIGGFGFTGQHNPIAYNSPYSQSIPSTAPGS*PR	6-phosphofructo-2-kinase		n/d
NCU03070	SLALNSGGPRS*PFPIDR	hypothetical protein		yes
NCU04164^2^	T*PTPGKYFGPPK	hypothetical protein		yes
NCU05041	IDEHDIARS*PGTVGLEETGSVDR	trehalose-phosphatase		yes
NCU05041	TESSLPGHLRPSVINVPVT*PGISR	trehalose-phosphatase		yes
NCU05041	TESSLPGHLRPS*VINVPVTPGISR	trehalose-phosphatase		yes
NCU06247^2^	TAAS*NETTSREAT*PR	hypothetical protein		yes
NCU06247^2^	LEVPHS*PR	hypothetical protein		yes
NCU07868^2^^,^ 4	TPSSTATPDS*PR	hypothetical protein		yes
NCU07868^2^^,^ 4	SVEAPQPSAALQSLRS*AR	hypothetical protein		yes
NCU07868^2^^,^ 4	SVEAPQPS*AALQSLR	hypothetical protein		yes
NCU07868^2^^,^ 4	TST*PVSTPK	hypothetical protein		yes
**NCU01789** 3 **^,^** 4	HEVPRS*PDDAKVVDLFK	*hyphal anastomosis-5*	*ham-5*	**NO**

1 Phosphopeptides with MAPK site in either 1 or 2(*) experiments, p<0.05, 1.5× decreased after 1NM-PP1 treatment.

2 Showed reduced expression in Δ*pp-1* germlings (19).

3 Showed reduced expression in a *mak-2^Q100G^* germlings treated with 1NM-PP1 (19).

4 Proteins with a predicted MAPK docking motif (R/K-R/K-(X)_1-5_-I/L-X-I/L).

To identify potential direct MAK-2 targets, we inspected the 96 phosphopeptides that showed reduced abundance in the 1NM-PP1 treated *mak-2^Q100G^* germlings for MAPK consensus phosphorylation sites (P-X-S*/T*-P) [36], [37]. Nine proteins were identified, of which five were annotated as hypothetical proteins. One of the proteins was ASL-1, the transcription factor that is a target of the OS-2 pathway and which shows an ascospore-lethal phenotype [35]; Δ*mak-2* mutants also show an ascospore-lethal phenotype [8], [38]. The remaining proteins included a predicted 6-phosphofructo-2-kinase (NCU01728), a predicted trehalose phosphatase (NCU05041) and a protein previously reported to be required for hyphal fusion, HAM-5 [23], [29] (Table 1). In addition to a predicted MAPK phosphorylation site, a predicted MAPK docking motif (R/K-R/K-(X)_1-5_-I/L-X-I/L) [39] was predicted in NCU07868 (hypothetical protein) and HAM-5 (Table 1). Of the nine genes encoding potential MAK-2 phosphorylation targets, five showed a reduction in expression levels in either a Δ*pp-1* mutant (transcription factor that is a target of the MAK-2 pathway) or in a *mak-2^Q100G^* germlings treated with 1NM-PP1 [24], including *ham-5* (Table 1).

To determine whether the putative direct or indirect MAK-2 phosphorylation targets were involved in germling fusion, strains carrying individual deletions of 8 out of the 9 genes whose proteins contained a MAPK consensus phosphorylation site and showed decreased abundance in the 1NM-PP1-treated *mak-2^Q100G^* germlings (including strains carrying deletions in all five hypothetical proteins) (Table 1), plus an additional 31 deletion mutants selected from a subset of proteins that showed decreased abundance in 1NM-PP1-treated *mak-2^Q100G^* germlings, but which lacked a predicted MAPK consensus phosphorylation site (Table S1), were evaluated for the ability to undergo chemotropic interactions and cell fusion. Of these, Δ*ham-5* in the first set, and Δ*ham-9*, Δ*ham-11*, and Δ*mak-1* strains in the second set, were fusion defective.

### Putative MAK-2 target HAM-5-GFP localizes in an oscillatory manner to cell tips during chemotrophic interactions

**Figure 2:**
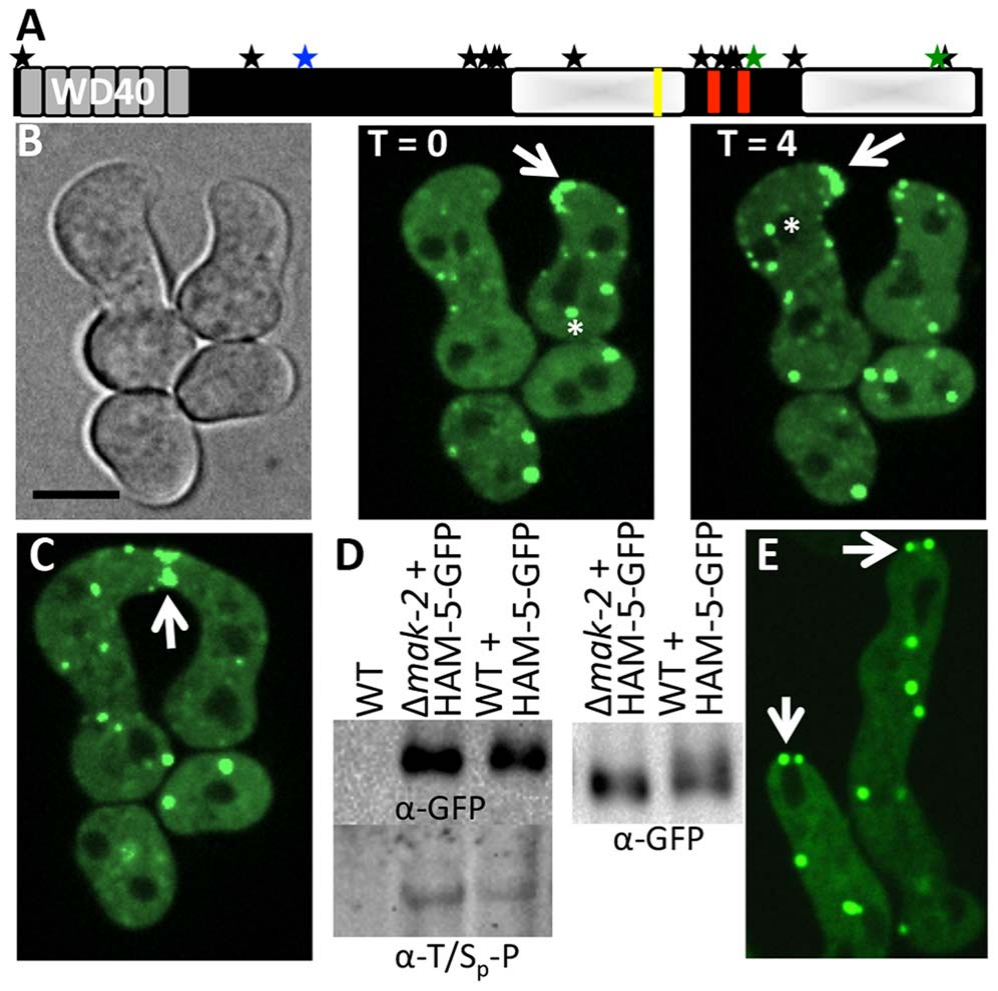
HAM-5-GFP localization in WT and Δ*mak-2* germlings. (A) Schematic overview of HAM-5 protein structure. The predicted WD40 domains are shown in grey and the putative coiled coil domains are shown as red bars. The two disordered regions with low complexity are depicted by shaded white boxes. The MAPK phosphorylation site (aa 506) is marked by a blue star, the other two sites showing decreased abundance in treated cells (aa 1288 and 1604) are marked by green stars, and other 13 identified phosphorylation sites (S14, S414, S792, S818, S833, T838, T969, S1085, S1199, T1201, S1202, T1353, S1608) [40] are marked by black stars. The putative MAPK docking site is marked by a yellow line. (B) Localization of HAM-5-GFP to puncta localized to CAT tips during chemotropic interactions between genetically identical cells. HAM-5-GFP showed dynamic localization to CAT tips of germlings with an oscillation of every four min (arrow). HAM-5-GFP also localized to puncta within germlings and near nuclear compartments devoid of HAM-5-GFP (asterisks). The image left is a bright field image. Scale bar  = 10 µM. (C) HAM-5-GFP localized to the sites of contact during germling fusion (arrow). (D) Western blots of WT, WT (*ham-5-gfp*) and Δ*mak-2* (*ham-5-gfp*) germlings with immunoprecipitated HAM-5-GFP probed with anti-GFP antibodies (right panel shows longer run showing higher mobility of HAM-5-GFP in wild type germlings) specifically detecting HAM-5-GFP (210 kD; Figure S4D). Lower panel shows a Western blot with identical samples probed with anti-phospho antibodies that specifically detect phosphorylated serine or threonine residues followed by a proline. (E) Localization of HAM-5-GFP to puncta in Δ*mak-2* germlings. Some puncta showed localization to germling tips, but which did not oscillate during growth (white arrows). Scale bar  = 10 µM.

To further characterize putative MAK-2 targets that are required for germling fusion, we GFP-tagged proteins where localization during chemotropic interactions/cell fusion had not been determined. This effort included HAM-5. The *ham-5* mutant was identified in a forward screen for mutants that failed to form a heterokaryon [29] and encodes a large protein of 1686 amino acids (aa) with seven putative WD40 repeats at the N-terminus (aa 14-313, grey boxes; Figure 2A). At the C-terminus, an unstructured region of low complexity was identified (shaded white boxes; Figure 2A) that contains stretches of predominately proline and glutamine residues. Two coiled coil domains were also predicted in HAM-5 (aa 1168-1190 and 1257-1286, red boxes; Figure 2A). In total, 16 phosphorylation sites were identified, located mainly at the middle section of HAM-5 (Figure 2A). Three phosphorylation sites were identified from our phosphoproteomics analysis, one of which was a putative MAPK site at amino acid residue 506 (serine) (Figure 2A; Table 1). Thirteen additional HAM-5 phosphopeptide sites were identified in a recent phosphoproteomics study of *N. crassa* hyphal cultures exposed to different carbon sources [40]. A putative MAPK docking site was also identified between residues 1128-1136 (RRKPPALDL) in the C-terminus of HAM-5 (yellow bar; Figure 2A).

In conidia, germlings and in hyphae, HAM-5-GFP fluorescence was observed in small, cytoplasmically localized puncta (white arrows, Figure S2). In mature hyphae, localization to septa was also observed (red arrow, Figure S2A). During chemotropic interactions between germlings, HAM-5-GFP driven by the *tef-1* promoter was observed as small puncta in interacting germlings and also at the tip (Figure 2B). Importantly, HAM-5 oscillated to CAT tips in germling pairs during chemotropic interactions, with localization dynamics similar to MAK-2 or SO: HAM-5-GFP appeared at the CAT tip of one germling, but was absent from the CAT tip in the partner germling, while 4 min later, HAM-5-GFP was present at the CAT tip of the second partner germling, but was absent from the CAT tip of the first germling (Movie S1). During the fusion process, a HAM-5-GFP signal was also observed in puncta that localized either to the cell periphery or to points close to nuclear compartments, which were devoid of HAM-5-GFP (Figure 2B, asterisk). When HAM-5-GFP localized to the CAT tips, the number of cytoplasmically localized puncta in the partner germling was reduced and the GFP signal was more dispersed in the cytoplasm (Figure 2B). HAM-5-GFP was detected at the site of germling contact and remained there until the cytoplasm of the two germlings mixed (Figure 2C).

To assess whether the HAM-5-GFP-tagged versions were biologically functional, we crossed wild type (WT) strains carrying *ham-5-gfp* driven by either the native, *tef-1* or *ccg-1* promoter into the Δ*ham-5* mutant. Progeny bearing both *ham-5-gfp* and Δ*ham-5* showed a fusion phenotype and frequency similar to WT, indicating that *ham-5-gfp* driven either by the native, *ccg-1* or *tef-1* promoter was functional. Localization of HAM-5-GFP driven by either *ccg-1* or *tef-1* in the Δ*ham-5* strain was similar to HAM-5-GFP in the WT strain, and showed localization to puncta and oscillation to CAT tips during chemotropic interactions. *ham-5-gfp* driven by its native promoter in the Δ*ham-5* strain also showed localization to puncta (Figure S3). However, due to low expression levels of HAM-5-GFP in this strain, localization during chemotropic interactions could not be fully assessed. In the following sections, HAM-5-GFP is shown driven by the *tef-1* promoter unless stated otherwise. A heterokaryon of a strain carrying *ham-5-gfp* driven by *tef-1* and a strain carrying histone *H1-dsRED* (a nuclear marker), showed non-overlapping fluorescence (Figure S2), indicating that HAM-5-GFP was excluded from the nucleus. This localization pattern is similar to SO-GFP [19], but different than MAK-2-GFP, which localizes to the cytoplasm and to nuclei (Figure S2) [19].

### MAK-2 regulates HAM-5 dynamics

HAM-5 is predicted to be a highly phosphorylated protein, with 16 predicted phosphorylation sites (Figure 2A). To assess whether HAM-5 is a phosphorylation target of MAK-2, as indicated by the phosphoproteomics data, we introduced *ham-5*-*GFP* into a *Δmak-2* strain and determined its phosphorylation status during germling fusion. HAM-5-GFP was immunoprecipitated from WT (*ham-5-gfp*) and *Δmak-2* (*ham-5-gfp*) 5 hr-old germlings using anti-GFP antibodies and assayed for phosphorylation status using Western blot analysis with anti-phosphoserine/threonine antibodies that specifically recognize phosphoserine or phosphothreonine sites followed by a proline residue (MAPK phosphorylation sites). The results showed that HAM-5-GFP from both WT and *Δmak-2* cells was specifically phosphorylated (Figure 2D). The HAM-5-GFP protein band from WT germlings showed a slight smear upwards and was slightly larger than that observed in Δ*mak-2* (*ham-5-gfp*) germlings (Figure 2D). These data support the phosphoproteomics data, which suggested a MAK-2-dependent modification of HAM-5 during germling fusion (Table 1). However, it is clear that HAM-5-GFP was also phosphorylated in the Δ*mak-2* mutant, suggested possible additional regulatory inputs into HAM-5 via phosphorylation by other proteins during conidial germination/germling fusion.

To assess whether the identified phosphosite in HAM-5 (serine 506) was required for HAM-5 function during chemotropic interactions and germling fusion, strains carrying site-directed mutations whereby serine 506 was altered to an alanine (phosphorylation impaired) or a glutamate (phosphorylation mimic) residue were evaluated: chemotropic behavior and cell fusion in germlings carrying the *ham-5^S506A^* or *ham-5^S506E^* mutations driven by the *ccg-1* promoter were indistinguishable from wild type germlings and fully complemented the growth phenotype of the Δ*ham-5* strain (Figure S4A). To assess whether mutations in the predicted MAPK docking site affected HAM-5 function, a strain was constructed where the first three amino acids of the MAPK docking site were changed to alanine (RRKPPALDL to AAAPPALDL). However, germlings bearing this *ham-5* allele (*ham-5^RRK1128AAA^*) also showed a similar communication and fusion phenotype to WT germlings resulting in similar growth phenotype to WT and complemented fully the growth phenotype of the Δ*ham-5* strain (Figure S4A). Thus, the predicted MAPK docking site in HAM-5 might not be functional or additional MAPK docking sites are present making this site redundant for function.

We predicted that HAM-5-GFP would show altered localization when introduced into Δ*mak-2* germlings, due to the inability of these cells to undergo chemotropic interactions. However, HAM-5-GFP localized to puncta in Δ*mak-2; ham-5-gfp* germlings, as observed in wild type cells (Figure 2E). However, no oscillation of HAM-5-GFP puncta to cell tips was observed, consistent with the lack of fusion and chemotropic interactions in Δ*mak-2* germlings. HAM-5-GFP puncta were localized randomly within the cell, with some puncta close to the nuclear periphery and membrane, but cells also contained at least one HAM-5-GFP puncta localized to the cell tip (Figure 2E). The stable, tip-anchored localization of HAM-5-GFP (Figure 2E, arrows) was not observed in WT germlings in the absence of chemotropic interactions (in isolated germlings), and was unique to Δ*mak-2; ham-5-gfp* cells.

### HAM-5-GFP oscillates with the MAP kinase cascade members to CAT tips

**Figure 3:**
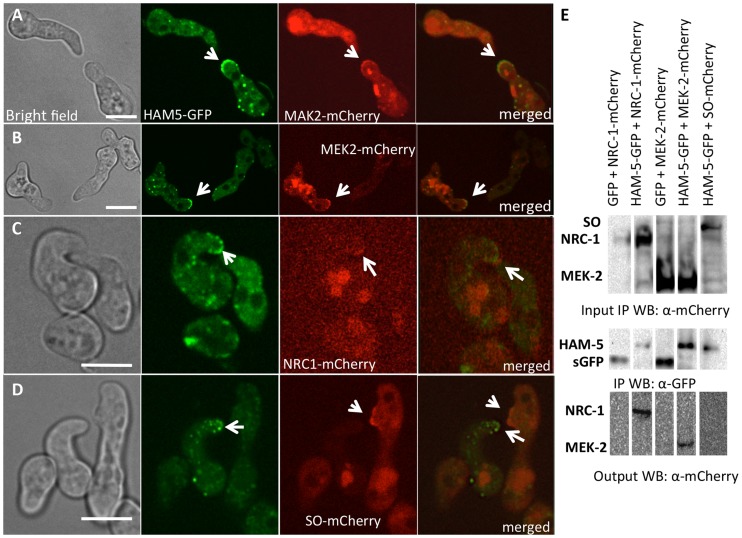
HAM-5-GFP shows localization with components of the MAK-2 pathway. (A) Co-localization of HAM-5-GFP and MAK-2-mCherry during germling communication (arrows). (B) Co-localization of HAM-5-GFP and MEK-2-mCherry during germling communication (arrows). (C) Co-localization of HAM-5-GFP and NRC-1-mCherry during germling communication. NRC-1-mCherry strains show low fluorescence [18]. (D) HAM-5-GFP and SO-mCherry do not co-localize during chemotropic interactions, but instead show opposite localization to CAT tips in communicating germlings (arrows). The images on the left are bright field images, fluorescent images on the right. Scale bar  = 10 µM. (E) Co-immunoprecipitation experiments showing an interaction between HAM-5-GFP (210 kD; Figure S4D) and MEK-2-mCherry (82.9 kD; Figure S4C) and NRC-1-mCherry (128 kD; Figure S4C). Input panels show Western blots of immunoprecipitated protein samples from 5 hr-old germlings probed with either anti-GFP (free GFP  = 27 kD; Figure S4D) or anti-mCherry antibodies. The output panel is a Western blot of proteins immunoprecipitated by anti-GFP antibodies (and thus HAM-5-GFP) and probed with anti-mCherry antibodies (detecting MEK-2-mCherry or NRC-1-mCherry).

The observation that HAM-5-GFP showed oscillation during germling fusion, but still localized to puncta in Δ*mak-2* germlings, suggested that either HAM-5 interacted with SO or with other proteins in the MAK-2 signal transduction pathway. Previously, it was shown that the components of the MAK-2 pathway, the MAPKKK (NRC-1) and the MAPKK (MEK-2), oscillate with MAK-2 during chemotropic interactions in germlings [18]. To determine if HAM-5 oscillated with the components of the MAK-2 complex or with SO, we used heterokaryons of strains carrying *ham-5-gfp* and either mCherry-tagged *mak-2*, *mek-2*, *nrc-1* or *so* alleles and examined co-localization of these proteins during chemotropic interactions in germlings. As shown in Figure 3, HAM-5-GFP + MAK-2-mCherry, or HAM-5-GFP + MEK-2-mCherry or HAM-5-GFP + NRC-1-mCherry were co-recruited to the CAT tips during chemotropic interactions. In homokaryotic strains carrying both HAM-5-GFP and MAK-2-mCherry, the dynamics of the two proteins were identical and showed simultaneous oscillatory recruitment to CAT tips (Movie S2). HAM-5-GFP + MAK-2-mCherry and HAM-5-GFP + MEK-2-mCherry also co-localized to cytoplasmic puncta during chemotropic interactions (Figure 3). As observed in [18], the NRC-1-mCherry signal was weak, and recruitment of HAM-5-GFP + NRC-1-mCherry to cytoplasmic puncta could not be assessed. In contrast to MAK-2/MEK-2/NRC-1, SO-mCherry + HAM-5-GFP always appeared at opposite CAT tips (Figure 3D) and with exactly opposite dynamics during chemotropic interactions (Movie S3).

**Figure 4:**
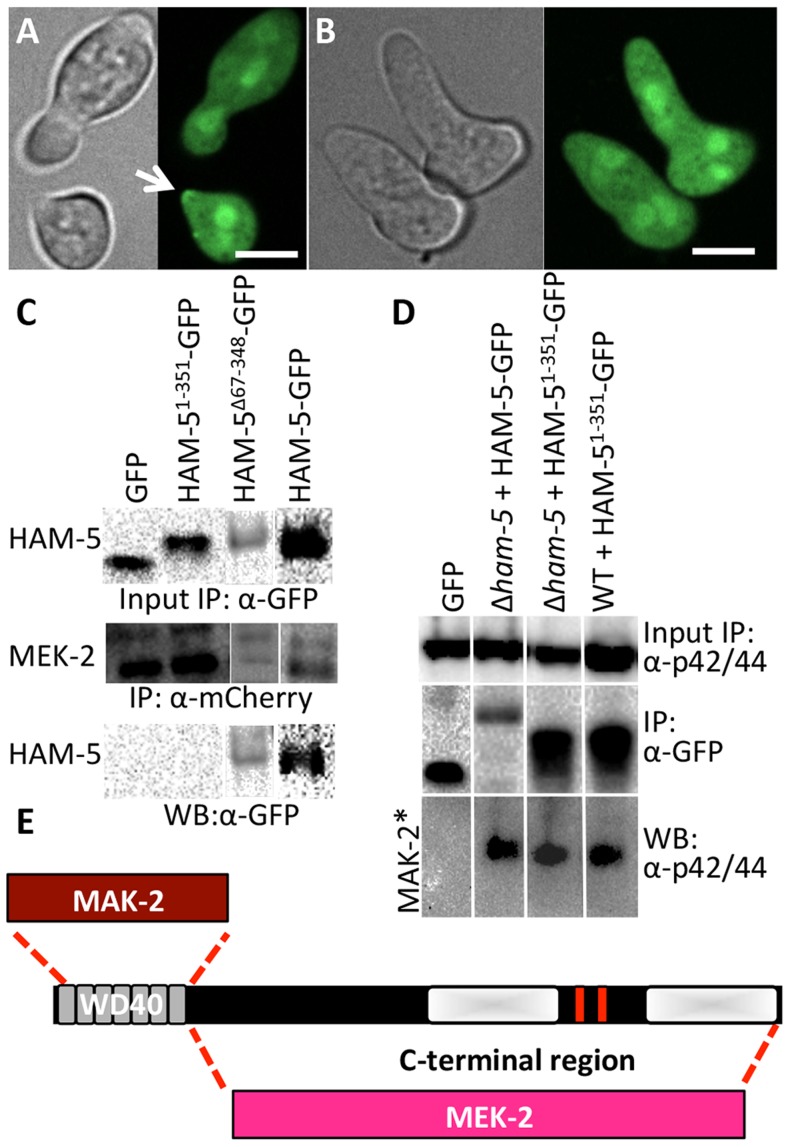
The WD40 domain of HAM-5 interacts with MAK-2, while the C-terminus interacts with MEK-2. (A) Localization of HAM-5^1-351^-GFP (WD40 domain only) in WT germlings localized to the cytoplasm, the nucleus and the puncta at the cell periphery and at the tip during chemotropic interactions and oscillation (arrow). (B) HAM-5^1-351^-GFP in Δ*ham-5* germlings localized to the cytoplasm and the nucleus; no puncta were observed. The images on the left are bright field images. Scale bar  = 10 µM. (C) Western blots showing a specific interaction between MEK-2-mCherry (82.9 kD) with full length HAM-5-GFP (210 kD) or HAM-5^Δ67-348^-GFP (180 kD), but not with free GFP (27 kD; Figure S4C, D). An interaction between MEK-2-mCherry and HAM-5^1-351^-GFP (65.3 kD) was not detected. Input panels show Western blot of immunoprecipitated proteins isolated from 5 hr-old germlings probed with either anti-mCherry antibodies or anti-GFP antibodies. Output panel shows anti-mCherry immunoprecipitated proteins (MEK-2-mCherry) probed with anti-GFP antibodies (HAM-5-GFP). (D) Western blots showing a specific interaction between full length HAM-5-GFP or HAM-5^1-351^-GFP and phosphorylated MAK-2. Input panels show Western blot of proteins isolated from 5 hr-old germlings probed with anti-p42/44 antibodies (which recognize phosphorylated MAK-2 (40.8 kD; Figure S4D, E) [8]) or immunoprecipitated proteins probed with anti-GFP antibodies. The output panel shows anti-GFP immunoprecipitated protein sample probed with anti-p42/44 antibodies, showing interaction between HAM-5-GFP or HAM-5^1-351^-GFP and phosphorylated MAK-2. (E) Schematic showing regions of interaction between HAM-5 and MAK-2 or MEK-2 based on co-immunoprecipitation experiments.

The localization of HAM-5 during chemotropic interactions suggested that HAM-5 physically interacts with MAK-2, MEK-2 and/or NRC-1. To test this hypothesis, we performed co-immunoprecipitation experiments using strains carrying HAM-5-GFP + MAK-2-mCherry, HAM-5-GFP + MEK-2-mCherry or HAM-5-GFP + NRC-1-mCherry. As controls, we used strains carrying MAK-2-mCherry, MEK-2-mCherry or NRC-1-mCherry-tagged proteins with GFP driven by the *ccg-1* promoter; GFP in these strains showed only cytoplasmic localization and was never observed in puncta. A specific interaction between HAM-5-GFP and MEK-2-mCherry and HAM-5-GFP and NRC-1-mCherry was detected when HAM-5-GFP was immunoprecipitated using anti-GFP antibodies from 5 hr-old germlings and subsequently re-probed using anti-mCherry antibodies (Figure 3E), consistent with the co-localization of these proteins observed by confocal microscopy (Figure 3A–C). No interaction between the mCherry-tagged proteins and cytoplasmic GFP was observed (Figure 3E; Figure S4C). An interaction between MAK-2-mCherry and HAM-5 could not be assessed, as MAK-2-mCherry showed non-specific binding. However, using phospho-specific anti-P42/P44 (Erk1/Erk2) antibodies that recognize phosphorylated MAK-2 [8], an interaction between HAM-5-GFP and MAK-2 was detected via co-immunoprecipitation (Figure 4D). By contrast, no interaction was detected between HAM-5-GFP and SO-mCherry (Figure 3E).

### The HAM-5 WD40 domain is required for function and stability and binds to MAK-2 but not to MEK-2

HAM-5 is a large protein with predicted protein-protein interaction domains including seven WD40 repeats, which are predicted to form β-propeller structures that have been implicated in coordinating protein assemblages in other systems [41]. To test the hypothesis that the WD40 domain is involved in HAM-5-MAK/MEK-2/NRC-1 interactions, we constructed two mutant *ham-5* alleles: one in which the WD40 motifs (aa 67-348) were removed (HAM-5^Δ67-348^) and one in which only the first 351 aa including the WD40 motifs of HAM-5 were retained (HAM-5^1-351^). Both alleles were tagged with *gfp*, and function and localization were assessed in both WT and Δ*ham-5* mutant strains.

The *ham-5* ^1-351^ construct failed to complement the growth or fusion defects of the Δ*ham-5* mutant (Figure S4A). When observed microscopically, the localization of the HAM-5^1-351^-GFP in Δ*ham-5* germlings was cytoplasmic and nuclear; no puncta were observed (Figure 4B; Figure S5A). This result is in contrast to the full length HAM-5-GFP, which localized to puncta and was excluded from the nucleus (Figure 2; Figure S2). However, in a WT background, a portion of the HAM-5^1-351^-GFP localized to puncta and showed oscillation during chemotropic interactions between germlings (Figure 4A). These observations suggest that in WT germlings, HAM-5^1-351^-GFP may bind the native untagged HAM-5, resulting in localization to puncta when the complex oscillates to CAT tips during chemotropic interactions. To determine whether HAM-5^1-351^-GFP (in a Δ*ham-5* mutant) physically interacted with MAK-2 or MEK-2, we performed co-immunoprecipitation experiments using either anti-mCherry antibodies (for MEK-2-mCherry) or anti-p42/44 antibodies for MAK-2 [8]. HAM-5^1-351^ specifically immunoprecipitated phosphorylated MAK-2 but not MEK-2-mCherry (Figure 4C, D; Figure S4D).

The *ham-5^Δ67-348^* construct also failed to complement the growth or fusion defects of the Δ*ham-5* mutant (Figure S4A), consistent with an essential role for the HAM-5 WD40 domain. Cellular fluorescence of HAM-5^Δ67-348^-GFP in germlings or hyphae was not observed and less protein was produced than other GFP-tagged proteins (Figure S4B), suggesting that the WD40 domain is required for HAM-5-GFP stability. However, co-immunoprecipitation experiments revealed a specific interaction between HAM-5^Δ67-348^ and MEK-2-mCherry, but not with MAK-2 (Figure 4C; Figure S4F). These biochemical interaction studies indicated that the WD40 domain of HAM-5 is important for interactions with MAK-2 and the C-terminus is important for interactions with MEK-2. The predicted MAPK docking site, which was not required for function by mutational analyses (see above), is located in the C-terminus of HAM-5, indicating that this site is not essential for MAK-2-HAM-5 interactions.

### HAM-5 is required for assembly of the MAPK-cascade members MAK-2 and MEK-2 into puncta

**Figure 5:**
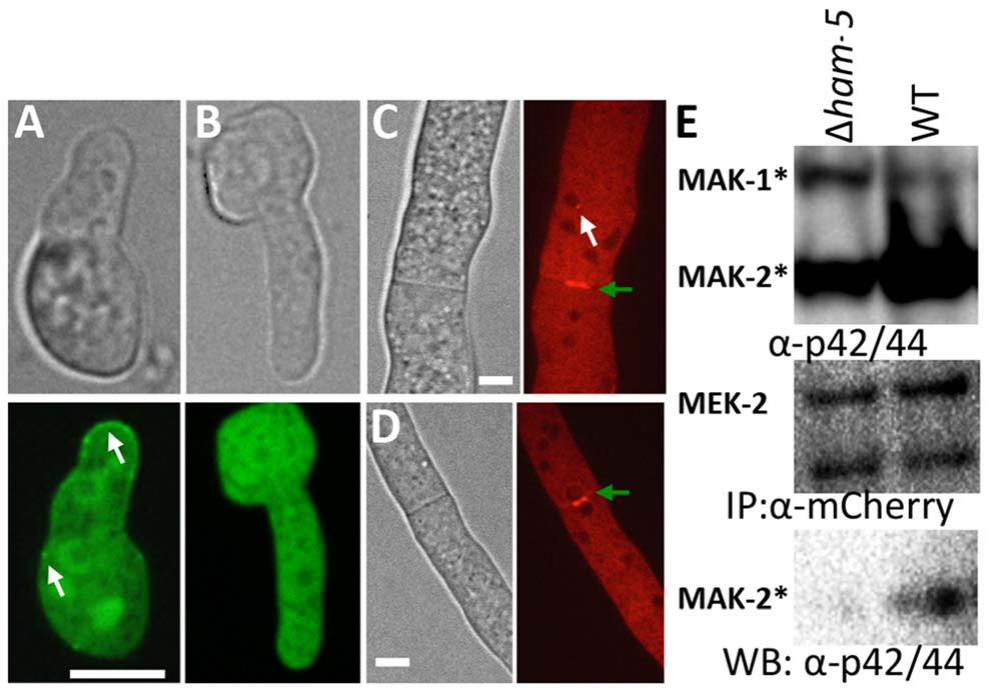
HAM-5 is required to localize MAK-2 and MEK-2 to puncta. (A) MAK-2-GFP in isolated WT germlings localizes to the nucleus, cytoplasm and to puncta (white arrows). (B) In the Δ*ham-5* mutant, MAK-2-GFP localization is cytoplasmic and nuclear; no puncta are observed. Scale bar  = 10 µM. (C) In WT hyphae, MEK-2-mCherry localizes to the septum (green arrow) and also to cytoplasmic puncta (white arrow). Scale bar  = 10 µM. (D) In Δ*ham-5* hyphae, septum localization of MEK-2-mCherry is observed (green arrow), but puncta are not. Scale bar  = 10 µM. (E) Co-immunoprecipitation experiments showing an interaction between MEK-2-mCherry and phosphorylated MAK-2 in WT (*mek-2-mCherry*) germlings, but a significant reduction in interaction between MAK-2 and MEK-2-mCherry in a Δ*ham-5* (*mek-2-mCherry*) strain. Top panel is a Western blot of protein samples probed with anti-p42/44 antibodies (MAK-2, 40.8 kD, MAK-1, 46.7 kD; Figure S4E). Middle panel is anti-mCherry immunoprecipitated proteins probed with anti-mCherry antibodies (MEK-2-mCherry, 82.9 kD; Figure S4C, E). Bottom panel is anti-mCherry (MEK-2-mCherry) immunoprecipitated proteins probed via Western blot with anti-p42/44 antibodies that recognize phosphorylated MAK-2.

Scaffold proteins such as Ste5 in *Saccharomyces cerevisiae* [42], which assembles the pheromone response MAPK pathway, regulates spatial functionality of this pathway via nuclear/plasma membrane shuttling of Fus3 during mating. We hypothesized that HAM-5 was also required for the assembly of the MAK-2 cascade members in complexes and subsequent recruitment of these complexes to their correct cellular location during chemotropic interactions (e.g. the CAT tip). To test this hypothesis, we introduced MAK-2-GFP or MEK-2-mCherry into a Δ*ham-5* strain. In contrast to WT and Δ*mak-2* germlings where HAM-5-GFP was localized to puncta (Figure 2), MAK-2-GFP showed cytoplasmic and nuclear localization in the Δ*ham-5* mutant; no puncta were observed (Figure 5A, B). In Δ*ham-5* hyphae, MEK-2-mCherry localized to the cytoplasm and to septa, but puncta were not observed as in WT hyphae (Figure 5C, D; Movie S4). We then tested whether HAM-5 was required to establish a stable interaction between MEK-2 and MAK-2. When MEK-2-mCherry was immunoprecipitated from WT germlings, phosphorylated MAK-2 was also detected, while in Δ*ham-5* germlings, co-immunoprecipitation of phosphorylated MAK-2 with MEK-2-mCherry was not detectable (Figure 5E; Figure S4E). SO-GFP was also cytoplasmically localized in Δ*ham-5* (*so-gfp*) germlings (Figure S5), a localization pattern identical to that observed in WT (*so-gfp*) germlings not undergoing chemotropic interactions.

**Figure 6:**
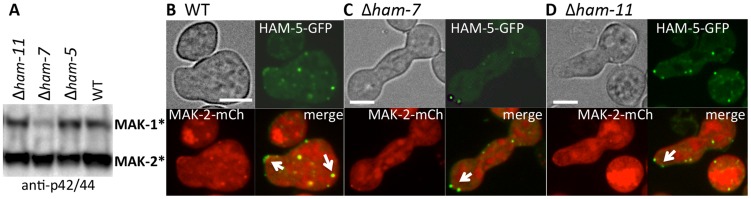
HAM-5-GFP and MAK-2-mCherry localize to puncta in Δ*ham-7* and Δ*ham-11* fusion-deficient germlings. (A) Western blot of protein samples from 5 hr-old WT, Δ*ham-5*, Δ*ham-7* and Δ*ham-11* germlings probed with anti-p42/44 antibodies, which recognize phosphorylated MAK-1 and MAK-2 [8]. As previously shown [43], MAK-1 phosphorylation is reduced in the Δ*ham-7* mutant. (B) HAM-5-GFP and MAK-2-mCherry localization in WT germlings undergoing chemotropic interactions. Arrows show localization to the CAT tip and to cytoplasmic puncta. (C) HAM-5-GFP and MAK-2-mCherry co-localization in Δ*ham-7* (*ham-5-gfp; mak-2-mCherry*) germlings. Note lack of chemotropic interactions. Arrows show puncta of HAM-5-GFP and MAK-2-mCherry in Δ*ham-7* (*ham-5-gfp; mak-2-mCherry*) germlings, which are often co-localized at the germ tube tip. (D) HAM-5-GFP and MAK-2-mCherry co-localization in Δ*ham-11* (*ham-5-gfp; mak-2-mCherry*) germlings. Note lack of chemotropic interactions. HAM-5-GFP and MAK-2-mCherry co-localized to both cytoplasmic and tip-localized puncta (arrows). The upper left panels are bright field images, the upper right panels show GFP fluorescence, the lower left panels show mCherry fluorescence. Lower right panels show merged images of GFP and mCherry images. Scale bars  = 10 µM.

Phosphorylation of MAK-2 by the upstream kinase, MEK-2, is required for fusion [18], but is not fully dependent on functional HAM-5. In a Δ*ham-5* mutant, phosphorylated MAK-2 is still detectable in hyphal preparations [29]. We confirmed this result in germlings, where phosphorylated MAK-2 was also observed in the Δ*ham-5* samples (Figure 6A). MAK-2 phosphorylation is also observed in two other fusion mutant strains: Δ*ham-7* and Δ*ham-11* [24], [43] (Figure 6A). To investigate whether the localization of MAK-2 complexes to puncta was dependent on HAM-5 or the ability to undergo chemotropic interactions, we expressed HAM-5-GFP and MAK-2-mCherry in Δ*ham-7* and Δ*ham-11* cells. Although Δ*ham-7* and Δ*ham-11* germlings are unable to undergo chemotropic interactions and cell fusion [23], [24], [43], co-localization of HAM-5-GFP and MAK-2-mCherry to puncta was still observed (Figure 6B-D). Interestingly, as seen in the Δ*mak-2; ham-5-gfp* strain, tip-anchored HAM-5-GFP and MAK-2-mCherry were present in Δ*ham-7* and Δ*ham-11* germlings (Figure 6, arrows).

### HAM-5-GFP oscillates with MAK-2 during hyphal fusion

**Figure 7:**
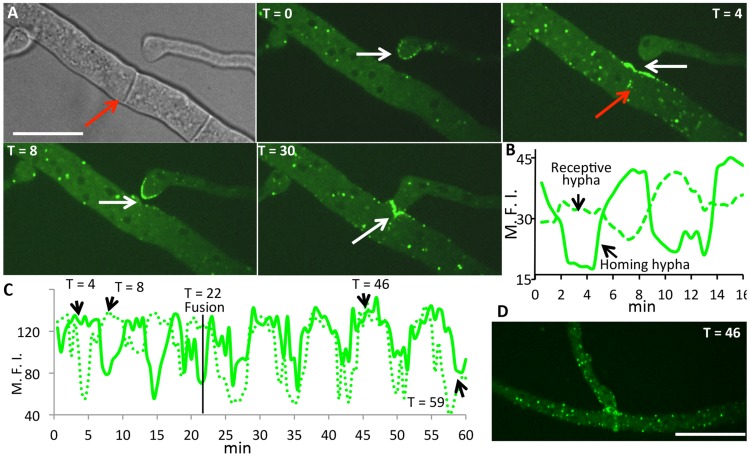
HAM-5-GFP shows oscillatory localization to fusion points and puncta in hyphae showing chemotropism. (A) Time course of HAM-5-GFP localization to interacting hyphae prior to cell fusion. HAM-5-GFP localized to the hyphal tip of a homing hyphae (white arrow T = 0; T = 8), followed by a disappearance and localization of HAM-5-GFP at the cell surface in the receptive hypha (white arrow; T = 4). Red arrow shows localization to septa near fusion points. At T = 30, HAM-5 is observed at the site of contact (white arrow). Bright field image is shown in upper left panel; remaining panels show GFP fluorescence. Scale bar  = 50 µM. (B) Graphical representation of relative fluorescence intensity (R.F.I.) of HAM-5-GFP localization to the receptive hypha and the homing hypha over the time course (panel A). × axis shows time (min). (C) Graphical representation of HAM-5-GFP fluorescence of interacting fusion hyphae shown in Figure S6B over an extended time course. Note that following the fusion event (T = 22 min), HAM-5-GFP puncta co-oscillate in both hyphae for an additional 30 minutes, see (D). *y* axis shows maximal fluorescence intensity (M.F.I.) while the × axis shows time (min). The time points at which the individual pictures taken at T = 4, T = 8, T = 46 and T = 59 minutes are pointed out in the graph (black arrows) (Figure S6 and Movie S5). The time of fusion at T = 22 min is marked with a black line. (D) Example of HAM-5-GFP appearing in puncta in both hyphae after fusion at T = 46.

Most mutants affected in germling fusion are also deficient in hyphal fusion [7], although localization of MAK-2 or SO during hyphal interactions has not been previously reported. Whether hyphal fusion is similarly coordinated as germling fusion is unknown, as different avoidance and fusion signals may be present at the periphery and older parts of a colony [44]. Another difference between germlings and hyphae is the presence of cytoplasmic flow in hyphae [10] that may influence the oscillation of proteins to sites of fusion. We therefore evaluated the localization of HAM-5-GFP during hyphal fusion in a mature colony. As shown in Figure 7, oscillation of HAM-5-GFP to the tips of hyphae undergoing chemotropic interactions was observed with dynamics very similar to that during germling fusion (where MAK-2 has a cycling time of ∼8 min at a single hyphal tip). Similar to germlings, MAK-2-mCherry also showed co-localization with HAM-5-GFP and oscillated with identical dynamics. MAK-2-mCherry was also observed in nuclei, while HAM-5-GFP was excluded (Figure S6A and Movie S5).

In addition to localization to sites at fusion tips of hyphae, puncta containing HAM-5-GFP and MAK-2-mCherry within adjoining hyphal compartments also showed oscillation (Figure 7 and Figure S6). Interestingly, upon membrane merger (t = 22 min), oscillation in both fusion hyphae was completely coordinated for an additional 30 minutes (Figure 7C,D, Figure S6 and Movie S5). We further assessed how far oscillation of HAM-5-GFP puncta extended in hyphal compartments that surrounded a fusion point. In filamentous fungi like *N. crassa*, hyphal compartments are delineated by septa, but septa contain a pore through which organelles, including nuclei, can move [45]. We observed coordinated oscillation of HAM-5 in over six hyphal compartments that were ∼100 µm from the point of fusion for a total distance of ∼200 µm (Figure S7). Hyphal compartments that showed different or no oscillation of HAM-5 distant from the point of fusion were delineated by septa (Movie S6). These data show that the oscillation of HAM-5-GFP in the hyphal network was coordinated over large distances surrounding a fusion point, but could be restricted by septa. Cytoplasmic flow, septal plugging and fusion events in nearby hyphae may also affect the oscillation of HAM-5 in compartments surrounding hyphal fusion points.

## Discussion

In this study, we show that HAM-5 functions as a scaffold protein for the MAK-2 MAP kinase complex and is required for oscillation of this complex during chemotropic interactions during germling and hyphal fusion in *N. crassa*. Our findings are complemented by the accompanying study of Dettmann *et al*., [46] that show physical interaction between HAM-5 and MAK-2/MEK-2/NRC-1 via mass spectrometry and yeast two hybrid, assessing both indirect and direct physical interactions of HAM-5 with the MAK-2 kinase complex. In other filamentous ascomycete species, mutations in *nrc-1, mek-2*, *mak-2* orthologs results in strains unable to undergo vegetative cell fusion [47], [48], [49], [50] as well as defects in growth, reproduction, virulence and host colonization phenotypes, indicating expanded functions for this MAPK pathway in filamentous fungi as compared to yeast [51], [52], [53], [54], [55], [56]. *ham-5* is highly conserved in the genomes of filamentous ascomycete species [51]. We predict that these *ham-5* homologs will function as a scaffold in these species for *mak-2/mek-2/nrc-1* orthologs, and which may be important for mediating growth, reproduction and virulence functions of this important and conserved signal transduction pathway.

The MAK-2 MAPK signal transduction pathway (MAK-2, MEK-2 and NRC-1) in filamentous fungi is orthologous to the pheromone response pathway in *S. cerevisiae* (Fus3, Ste7 and Ste11). Previously, it was shown that a *FUS3/KSS1* ortholog in the filamentous ascomycete species *Magnaporthe grisea*, (*PMK1*) as well as its ortholog in *Aspergillus nidulans* (*mpkB*) complements the pheromone response/mating defect of a *S. cerevisiae fus3Δ/kss1Δ* mutant [49], [57]. In *S. cerevisiae*, the Ste5 scaffold protein allosterically facilitates Ste7 phosphorylation of *N. crassa* MAK-2 (called *N. cra mpkB*) [58] and *A. nidulans* MpkB [57], indicating conservation of regulation of these kinases by allosteric motifs within Ste5. However, although components of these two MAPK pathways are highly homologous in fungi, an ortholog of *STE5* is absent in the genomes of filamentous ascomycete species. Future experiments comparing the function of these non-homologous scaffold proteins (STE5 and HAM-5) in regulating conserved signal transduction pathways will reveal how selection and evolution has shaped convergent evolution of these processes.

In *S. cerevisiae*, Gβγ is involved in the recruitment of Ste5 to the plasma membrane upon pheromone exposure, thereby recruiting the Fus3 MAPK cascade to the membrane. Membrane binding of Ste5 likely concentrates the bound MAP kinases spatially, promoting amplification of the signal [59], [60]. In *N. crassa*, how HAM-5 and MAK-2 are recruited to the membrane is still elusive, but is dissimilar from Ste5 since the Gβγ ortholog in *N. crassa* is not involved in germling fusion [61], [62]. Other upstream factors shared between *S. cerevisiae* and *N. crassa* might regulate the activation of the MAPK pathway. One is STE50, a component in yeast that helps to activate Ste11. In the accompanying article, Dettmann *et al*., [46] identified a role for *N. crassa* STE-50 as an activator of NRC-1; Δ*ste-50* mutants were fusion deficient. Three other proteins acting upstream of NRC-1 and STE-50 were also identified: the MAP4 kinase STE-20, the small GTPase RAS-2/SMCO-7 and the capping protein of the adenylate cyclase (AC) complex, CAP-1/NCU08008. Strains carrying a deletion of any of these three genes still showed residual germling fusion, suggesting multiple and redundant inputs into the MAK-2 signal transduction pathway. In *S. cerevisiae*, Bem1 interacts with Ste20, Ste5 and actin [63]. In *N. crassa*, strains carrying either a deletion of *bem-1*, encoding a predicted scaffold for NADPH oxidase (NOX), or its regulator (NOXR), are germling/hyphal fusion deficient [23]. Activated RAC-1 also localizes to CAT tips during chemotropic interactions and may also function as an upstream activator [64]; *Δrac-1* mutants are also are germling fusion defective [23].

**Figure 8:**
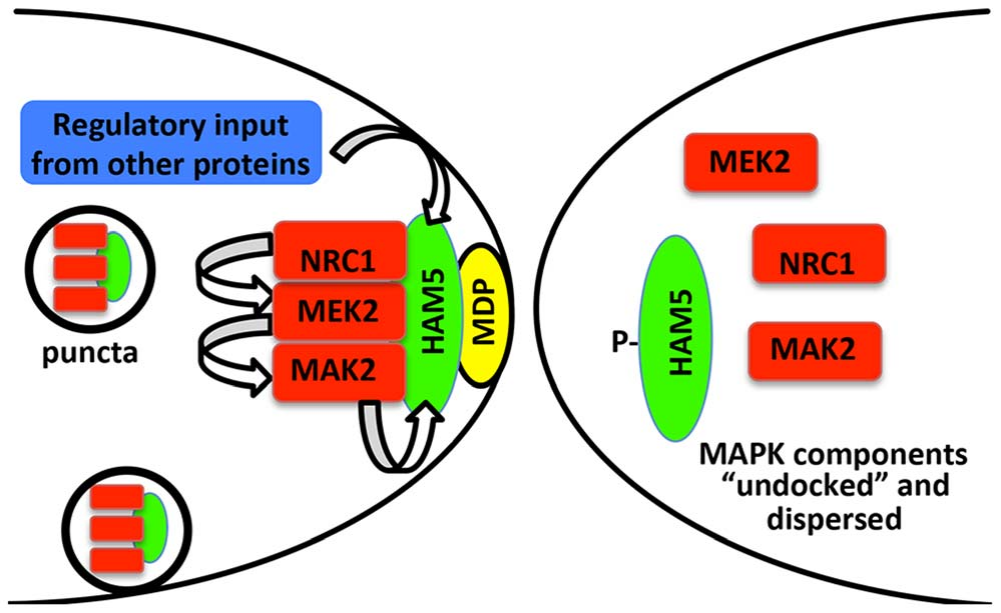
Model for HAM-5-MAK-2/MEK-2/NRC-1 function during chemotropic interactions. HAM-5 interacts with MAK-2, MEK-2 and NRC-1 and assembles in puncta throughout the germling, some of which are recruited to the CAT tip during chemotropic interactions via interactions with a plasma membrane associated protein (MDP: membrane docking protein). Association of the HAM-5/MAK-2/MEK-2/NRC-1 complex to the CAT tips is associated with signal reception from the partner germling. During this process, HAM-5 is successively phosphorylated by MAK-2 and other kinases, resulting in the disassociation of the complex and termination of the ability to receive signal. Nuclear MAK-2 signaling to the transcription factor PP-1 is not believed to be essential for chemotropic interactions as treatment of germlings with cycloheximide did not disturb oscillation of MAK-2 nor chemotropic behavior [19]. The coordinated assembly and disassembly of HAM-5/MAK-2/MEK-2/NRC-1 during communication regulates the tempo of chemotropic interactions between germlings.

During chemotropic interactions, HAM-5/MAK-2 complex assembles in puncta at the CAT tip and in the cytoplasm, followed by disassembly of HAM-5/MAK-2 complex from puncta, not just at the CAT tip, but from puncta observed throughout the germling and fusion hyphae, a cycle that repeats itself during chemotropic interactions every ∼8 min. In the absence of HAM-5, localization of MAK-2 kinase complex to puncta was impaired, while in Δ*mak-2* mutants, HAM-5 puncta were still observed. These observations indicate that MAK-2 kinase activity is essential for disassembly of the HAM-5/MAK-2 complex (Figure 8) during chemotropic interactions. The formation of HAM-5/MAK-2 complexes in puncta was not disrupted in other fusion mutants, such as Δ*ham-7* and Δ*ham-11*; cortical localization of the HAM-5/MAK-2 complexes were observed at cell tips, although oscillation was not. These data suggest that the HAM-5/MAK-2/MEK-2/NRC-1 complexes are poised to signal for chemotropic interactions, but that the absence of HAM-7 or HAM-11 disrupts signaling that results in disassembly of the HAM-5/MAK-2 complexes, both within the cell and at the cell cortex.

Few downstream targets of MAK-2 have been identified in filamentous fungi. The PP-1 protein, a transcription factor similar to Ste12 from yeast, is a likely downstream factor that is required for the activation of genes that play a role during the cell fusion and membrane merger [24], [46], [65]. Another target of MAK-2 is MOB-3, a protein of the STRIPAK complex involved in cell fusion that assures correct nuclear localization of MAK-1 [66]. Among the phosphorylated proteins in addition to HAM-5 identified in this study are members of the osmosensing (OS-2, CUT-1 and ASL-1) and cell wall integrity pathways (MAK-1) and other proteins of unknown functions but which are required for fusion (HAM-9 and HAM-11) (Table S1). The accompanying study [46] also identified MAK-1, OS-2 and CUT-1 in MAK-2 complexes via mass spectrometry. Both studies also identified other proteins that interacted with MAK-2/MEK-2/NRC-1 complex [46] or as potential phosphorylation targets of MAK-2 (this study; Table S1), including a glucokinase (NCU00575), SUC (pyruvate decarboxylase), an aminotransferase (NCU03500), a trehalose-phosphatase (NCU05041), CAMK-4 calcium/calmodulin-dependent kinase-4, and four hypothetical proteins (NCU00627, NCU00935, NCU006247 and NCU08330) [46]. The identification of MAK-2 phosphorylation targets provides new clues to the interconnectivity of signaling pathways in *N. crassa*; these two combined datasets will be a rich resource for further studies on the MAPK pathway function in fungi.

It is challenging to identify kinase targets that are often present in low abundance and have low phosphorylation stoichiometry, from a complex whole cell lysate with limited sample size. The multiplexed iTRAQ quantitation strategy, high specificity of phosphopeptide enrichment and high resolution nano-flow LC separation coupled to MS, as used in this study, have together contributed to the success of identifying and quantifying thousands of phosphopeptides from a small size sample (∼200 µg protein per sample condition). Our phosphoproteomics dataset from 5 hr old germlings provides information on stage-specific phosphorylation events on over ∼1100 proteins (Dataset S1). This dataset can be further compared to a recently published study on ∼3500 phosphorylated proteins identified under hyphal conditions and different carbon sources [40]. Both of these studies provide rich datasets for the filamentous fungal research community to interrogate the identity and function of phosphorylation sites on a large fraction of proteins in the *N. crassa* proteome. For example, three phosphorylation sites on HAM-5 from germlings were identified from this study, but an additional 13 HAM-5 phosphopeptides were identified in a sample from a 20 hr-old hyphal culture [40]. For chemotropic interactions, further studies on the additional phosphorylation sites and further dissection of the protein domains in the C-terminus of HAM-5 may explain how this scaffold protein itself is recruited to puncta, and may reveal additional binding partners for HAM-5. Such studies will elucidate the molecular mechanism and function of oscillation of the HAM-5/MAK-2/MEK-2/NRC-1 complex during chemotropic interactions. Understanding the molecular basis of germling/hyphal fusion in filamentous fungi provides a window into fungal language and communication and provides a paradigm for self-signaling mechanisms in multicellular eukaryotic species.

## Materials and Methods

### Molecular techniques and strain construction

Deletion strains used to screen for fusion mutants and strains constructed for this study are listed in Table S2. Strains were grown on Vogel's minimal medium (VMM) [67] (with supplements as required) and were crossed on Westergaard's medium [68]. Transformations and other *N. crassa* molecular techniques were performed as described [69] or using protocols available at the Neurospora home page at the FGSC (http://www.fgsc.net/Neurospora/NeurosporaProtocolGuide.htm).

To construct the *ham-5* alleles, PCR was performed with the restriction enzyme linkers included in the primer region. We amplified *ham-5* alleles using primers 1-5: *ham-5*FXbaI tttttctagaATGTCGGTCCCCGGACACA; *ham-5*RpacI aaaattaattaaGATCATCTCACTATGATGCAAC; *ham-5* WD40onlyR tttttaattaaGTTAGCAGGATGTTGAACGTTG; *ham-5*RWD40 tttatgcatatttaaaTCATGGTGGCAGCATACAATC; *ham-5*FWD40 tttatttaaatCCTGCTAACATGTTACCTCC; and cloned the fragments into pCR-Blunt vector (Invitrogen). For constructing the point mutations at the predicted phosphorylation site we used fusion PCR strategy with primers CGTCATCGGGGGCGCGCGGCACCTCATGGCG and GGTGCCGCGCGCCCCCGATGACGCGAAAGTTGT (S → A) and CGTCATCGGGCTCGCGCGGCACCTCATGGCG and GGTGCCGCGCGAGCCCGATGACGCGAAAGTTGT (S → E) together with the primers TTTGATGCATCACAATGCTGACC and TTAAGGGCCGAATTCTTCGC. The mutated constructs were ligated into the *Nsi*I and *Eco*RI sites of the *HAM-5* gene. For constructing the mutation at the predicted docking site, we used primers ggtcgctgacaaactcgaat and tttgccggctgcCTCGGAACTGCGCGCGCGG that has the restriction site *Nae*I in the linker and TTTCGGCCAGCATCATGAGA and tttagcgctCCTCCAGCACTCGACCTTCGC that has the restriction site *Afe*I in the linker. The two respective products were digested with *Nsi*I and *Nae*I and *Afe*I and *Tth*111I, respectively and ligated using a three-point ligation in the vector with *HAM-5* cut open with *Nsi*I and *Tth*111I.

We sequenced and digested the constructs from the pCR-Blunt vector with the appropriate restriction enzymes. The different fragments were ligated into plasmid pMF272 (AY598428) [70], [71]. For the MAK-2-mCherry, SO-mCherry, MEK-2-mCherry and NRC-1-mCherry strains, plasmid TSL84C was used. To generate plasmid pTSL84C, sGFP, from plasmid pMF272, was removed by digestion with *Pac*I and *Eco*RI restriction enzymes and replaced by a version of mCherry that is codon-optimized for *N. crassa* and includes a C-terminal 6x-His-tag (mCherryNc-6xHis). Plasmid pMFP26 [72] contains untagged codon-optimized mCherryNc and was used as a template for PCR using forward primer OTS177 (AAATTAATTAACGTGAGCAAGGGCGAGGAGGATAAC) and reverse primer OTS202 (AAAGAATTCCTAGTGGTGGTGGTGGTGGTGGCTGCCCTTGTACAGCTCGTCCATGCCGCCG), which contained information for the 6xHis tag and a stop codon. Plasmid derivatives with the *tef-1* promoter instead of *ccg-1* promoter were obtained by swapping the *ccg-1* promoter for the *tef-1* promoter using the restriction enzymes *Not*I and *Xba*I. A tandem construct of *tef-1-ham-5-gfp* and *tef-1-mak-2-mCherry* was created by digesting the *tef-1-mak-2-mCherry* pMF272 plasmid with restriction enzymes *Psp*OMI and *Bst*BI and the *tef-1-ham-5-gfp* construct with *Not*I and *Bst*BI. The latter fragment was ligated into the *tef-1-mak-2-mCherry* pMF272 plasmid to create *tef-1-mak-2-mCherry* and *tef-1-ham-5-gfp*. All constructs were transformed into the WT *his-3* strain with selection for His+ prototrophy. Homokaryotic strain was obtained via microconidial purification [73]. A strain bearing cytoplasmic GFP was obtained by transformation of the empty pMF272 plasmid into the WT *his-3* strain. All micrographs with HAM-5-GFP are with strains bearing the *tef-1-ham-5-gfp* constructs unless stated otherwise.

Deletion strains were obtained from the FGSC [74] that were generated as part of the *N. crassa* functional genomics project [69], [75]. For each deletion strain, both the mating type *A* and mating type *a* strains were analyzed, if available.

### Phenotypic analyses

To assess the ability of fusion between conidia of a deletion strain as compared to wild type, slant tubes containing the strains were grown for 4–6 days or until significant conidiation occurred. Conidia were harvested by vortexing the slant tube with 2 ml ddH_2_O and subsequently filtered by pouring over cheesecloth to remove hyphal fragments. Conidia were diluted to a concentration of 3.3×10^7^ conidia/ml. For each sample, 300 µl of spore suspension was spread on a 9 cm solid VMM plate. The plates were dried in a fume hood for 20-30 minutes and incubated for 3–4 hours at 30°C. Squares of 1 cm were excised and observed with a Zeiss Axioskop 2 using a 40× Plan-Neofluor oil immersion objective. The ability of germlings to communicate was determined by evaluating whether germlings displayed homing behavior when germinated conidia were within ∼15 um of each other.

### Phosphoproteomics sample preparation

Samples for protein extraction were grown for 4.5 h at 30°C and 200 rpm. Shaking was stopped and samples were grown for 20 minutes longer to encourage cell-cell interaction. The *mak-2^Q100G^* strain was treated with 10 µM 1NM-PP1 final concentration in DMSO or with DMSO alone for 10 minutes. Cells were harvested by filtration and frozen before protein extraction. Protein was extracted using the Trizol procedure (according to manufacture's protocols) and was kept in a solution of 6 M guanidine, 50 mM ammonium bicarbonate at pH 7.4. 200 µg for each sample was used for guanidine digestion: 1) pH was adjusted to pH 7.4 with the iTRAQ resuspending buffer (500 mM), 2) 1 hr reduction using 5 mM DTT at 56°C, 3) 1 h alkylation using 10 mM iodoacetamide at RT in the dark, 4) samples were diluted 10 fold with 25 mM NH_4_HCO_3_, pH 7.8, 5) 2 mM CaCl_2_ and trypsin was added at a 1∶50 (trypsin-to-protein) ratio and incubated for 3 hr at 37°C with gentle shaking, 6) trypsin was added again in the same ratio and samples were incubated over-night at 37°C with gentle shaking, 7) subsequently, a standard C18 Solid phase extraction (SPE) was performed with 80% ACN and no TFA, 8) samples were dried in a Speed-Vac and a bicinchoninic acid (BCA) assay was performed.

For 4-plex iTRAQ labeling, 100 µg lyophilized sample per iTRAQ label was used: 1) samples were reconstituted with 30.0 µL of dissolution buffer (500 mM triethylammonium bicarbonate), sonicated and vortexed to resuspend the peptides, 2) sample pH was checked (∼pH 8.5), 3) each vial of iTRAQ reagent (114, 115, 116 and 117) was brought to room temperature and 70 µL of ethanol was added to each iTRAQ reagent vial, 4) samples were vortexed for 1 min and spun down, 5) each labeled reaction mix was added to one separate sample, 6) samples were vortexed, spun down and incubated for 1 hr at RT. Samples were subsequently hydrolyzed by adding 300 uL of 0.05% TFA (3 times the volume), vortexed, spun down and incubated at room temperature for another 30 min, 7) samples were concentrated to 40 µL using a Speed-Vac, 8) samples were pooled into a fresh 2 mL silanized tube and concentrated to ∼100 µL using a Speed-Vac before a desalting (SPE C18) step was performed.

For phosphopeptide enrichment, magnetic Ni-NTA-agarose beads were obtained from Qiagen (Valencia, CA Part N#36111): 1) 50 µL of the 5% suspension metal ion activated NTA was used for 100 ug peptides, 2) beads were first prepared by washing 3× with nano-pure water (800.0 µL of water per 1.0 mL of bead suspension), 3) beads were then treated with 100 mM EDTA, pH 8.0 (800.0 µL of 100 mM EDTA per 1.0 mL of bead suspension) for 30 min with end-over-end rotation, 4) EDTA solution was removed, and beads were washed 3× with nano-pure water (at the same ratio). Subsequently, the beads were treated with 10 mM aqueous metal ion solution (800.0 uL of 100 mM FeCl_3_ per 1.0 mL of bead suspension) for 30 min with end-over-end rotation, 5) after removing excess metal ions, beads were washed 3× with water (at the same ratio), and resuspended in 1∶1∶1 acetonitrile/methanol/0.01% acetic acid for aliquoting into microcentrifuge tubes, 6) peptide samples were resuspended in 200.0 µl wash/resuspension buffer (80% acetonitrile, 0.1% TFA), 7) beads were washed with of 80% acetonitrile with 0.1% TFA (200 µL of 80% acetonitrile per 50.0 µL of beads) and precipitated using the magnetic stand; the supernatant was discarded. The resuspended samples (100 µg peptides in 200 µl of 80% acetonitrile, 0.1% TFA) were added to the activated beads and incubated for 30 min with end-over-end rotation, 8) beads were precipitated using the magnetic stand and washed for 1 min with 80% acetonitrile, 0.1% TFA. This step was repeated three more times, 9) phosphopeptides were eluted from the beads using an appropriate amount of elution buffer (50.0 µL of the elution buffer per every 50.0 uL of beads/100.0 ug of peptides) after incubating for 5 min, 10) the samples were then acidified to pH 4.0 by concentrating the samples down to 5−10 µl in a Speed-Vac and reconstituted in 30 µL with 0.1% TFA.

### Mass-spectrometry based analysis

All peptide samples were analyzed using an automated home-built constant flow nano LC system (Agilent) coupled to an LTQ Orbitrap Velos mass spectrometer (Thermo Fisher Scientific) operating in data-dependent mode [76]. Electrospray emitters were custom made using either 360 µm o.d. ×20 µm i.d. chemically etched fused silica. The nano LC system for phosphoproteomics analysis has an online 4-cm×360 µm o.d. ×150 µm i.d. C18 SPE column (5- µm Jupiter C18, Phenomenex, Torrence, CA) to desalt each phosphopeptide sample (20 µL), which is connected to a home-made 60-cm×360 µm o.d. ×50 µm i.d. capillary column (3- µm Jupiter C18, Phenomenex, Torrence, CA). Mobile phase flow rate was 100 nL/min and consisted of 0.1 M acetic acid in water and 0.1 M acetic acid in 70∶30 (v/v) acetonitrile:water. For each sample, three technical replicates of LC-MS analyses were performed as shown in Figure S1. These included (i) an LC gradient of 300 min with the LTQ Orbitrap Velos mass spectrometer acquiring higher-energy collisional dissociation (HCD) scans; (ii) an LC gradient of 300 min with the LTQ Orbitrap Velos mass spectrometer acquiring alternating collision-induced dissociation (CID), ETD (electron transfer dissociation), and higher-energy collisional dissociation (HCD) scans; (iii) an LC gradient of 180 min with the LTQ Orbitrap Velos mass spectrometer acquiring alternating collision-induced dissociation (CID), ETD (electron transfer dissociation), and higher-energy collisional dissociation (HCD) scans.

### Peptide identification and quantification

For peptide identification, MS/MS spectra were searched against a decoy *Neurospora* protein sequence database using SEQUEST [77]. Search parameters included: trypsin enzyme specificity with a maximum of two missed cleavages, +/- 50 ppm precursor mass tolerance, +/- 0.05 Da product mass tolerance, and carbamidomethylation of cysteines and iTRAQ labeling of lysines and peptide N-termini as fixed modifications. Allowed variable modifications were phosphorylation of serine, threonine or tyrosine residues. MSGF spectra probability value [78] was also calculated for peptides identified from SEQUEST search. Measured mass accuracy and MSGF spectra probability were used to filter identified peptides to <1% false discovery rate (FDR) at spectrum level.

iTRAQ reporter ions were extracted using the MASIC software [79] within 10 ppm mass tolerance of each expected iTRAQ reporter ion from each MS/MS spectrum. The sum of the individual iTRAQ reporter ion values from all MS/MS spectra for a given peptide was used for calculating their relative abundance across different conditions. To correct any systematic error due to pipetting, data were normalized by the median of iTRAQ reporter ion of the individual sample. Fold change of each phosphopeptide was calculated by dividing the data points from the two different conditions (i.e. control and 1NM-PP1-treated) and transformed into Log_2_ scale. Statistical analyses were performed using Students t-test. Only data with changes exceeding 1.5× greater in control versus 1NM-PP1-treated (p<0.05) were considered differential.

### Fluorescence microscopy

The strain used to cross *so-gfp* into a Δ*ham-5* strain was AF-SoT8 and the strain used to cross *mak-2-gfp* into a Δ*ham-5* strain was AF-M512 (Table S2).

Oscillation studies performed with HAM-5-GFP and mCherry tagged strains were prepared as described above with modifications from [20]. Images were taken using a Leica SD6000 microscope with a 100×1.4 NA oil-immersion objective equipped with a Yokogawa CSU-X1 spinning disk head and a 488-nm or 561-nm laser controlled by Metamorph software. Multiple pairs of interacting germlings were analyzed per experiment and representative pairs are shown for each strain. The ImageJ software was used for image analysis.

### Immunoprecipitations and western blotting

Harvested conidia (1×10^6^/ml) were inoculated in 100 ml VMM in flasks and incubated for 2.5 hrs at 30°C with shaking at 200 rpm, then an additional 2.5 hrs at 30°C without shaking. Germlings from 3 flasks were harvested by vacuum filtration over a nitrocellulose membrane and frozen in liquid nitrogen. Protein extraction from ground mycelium was performed using 1 ml lysis buffer described in [8] containing complete protease inhibitors, phosphatase inhibitor and Triton X-100. 20 µl supernatant was used for western blotting and the remaining fraction was used for immunoprecipitation using Protein G Dynabeads (Invitrogen), according to manufacturer's instructions, with the following exceptions: mouse or rabbit anti-GFP antibody (Roche or Life Technologies, respectively) or rabbit anti-mCherry antibody (Bio-vision) was covalently bound to the beads using BS^3^ (Sulfo-DSS, Fisher scientific) or DMP (dimethylpimelimidate) according to manufacturer's instructions.

Supernatant samples were incubated with the beads overnight at 4°C. Beads were washed with standard PBS for three times before protein was removed from the beads by heating at 70°C for 10 min in 1× loading buffer, and samples were run on a 4–12% Nu-Page Bis-Tris GelGel (NOVEX, Life Technologies). Protein gels were subjected to Western blot analysis using standard methods. Samples for the MAK-1 and MAK-2 phosphorylation western blots were treated similarly, except, after protein extraction with 1 ml lysis buffer, 25 µl of protein sample was directly loaded on a 7% NuPage Bis-Tris GelGel (NOVEX, Life Technologies) protein gel. Gels were subjected to Western blot analysis using standard methods and detection of phosphorylated MAK-1 and MAK-2 was carried out using anti-phospho p44/42 MAP kinase antibodies (1∶3000 dilution) (PhosphoPlus antibody kit; Cell Signaling Technology) as described [8]. Detection of phosphorylated HAM-5-GFP was performed using anti-phosphothreonine-proline/phosphoserine-proline antibodies (Abcam).

## Supporting Information

Figure S1 **Schematic overview of protein samples of DMSO (control) and 10 µM 1NM-PP1 in DSMO treated cells (two replicas each), downstream processing and mass spectrometry analysis.** Two flasks of control and 1NM-PP1 treated *mak-2^Q100G^* 5-hr old germlings were used to collect protein (left). Each of the four protein samples was digested with trypsin and subsequently treated with a barcoded iTRAQ label. The barcoded samples were mixed and subjected to immobilized metal affinity chromatography to enrich for phosphopeptides. The phosphopeptides were identified using liquid chromatography-mass spectrometry (LC-MS) with three different LC-MS acquisition methods. Statistical analyses were performed to identify peptides that differed in abundance between treatments.
(TIF)

Figure S2 **HAM-5-GFP localizes to puncta in conidia and mature hyphae, but is excluded from the nucleus.** (A) Localization of HAM-5-GFP to puncta in a hypha (white arrows) and to the septum (red arrow). The left panel shows a bright field image (scale bar  = 10 µM). (B) Localization of HAM-5-GFP to puncta in conidia (white arrows). The panel on the left shows a bright field image (scale bar  = 10 µM). (C) Composite of HAM-5-GFP and H1-dsRED during germling fusion. Upper left panel is a bright field image (scale bar  = 10 µM), upper right panel shows HAM-5-GFP fluorescence, lower left panel shows H1-dsRED fluorescence and lower right panel shows the composite of HAM-5-GFP and H1-dsRED fluorescent images. H1-dsRED localizes to the nucleus and is visible in vacuolar structures (lower left, white arrow and green arrows, respectively). (D) Composite of MAK-2-GFP and H1-dsRED during germling fusion. Upper left panels is a bright field image (scale bar  = 10 µM), upper right panel shows MAK-2-GFP fluorescence, lower left panel shows H1-dsRED fluorescence and the lower right panel shows the composite of MAK-2-GFP and H1-dsRED images.
(TIF)

Figure S3 **Localization of HAM-5-GFP in hyphae when driven by its native promoter.** Left panel: GFP fluorescence images showing HAM-5-GFP localization to puncta in hyphae (arrows). The right panel is a bright field image. Scale bar  = 10 µM.
(TIF)

Figure S4 **The WD40 domain is required for HAM-5 stability and function, but does not complement the Δ*ham-5* growth defect.** (A) Slant tubes with WT, Δ*ham-5*, Δ*ham-5*+ *ham-5-gfp*, Δ*ham-5*+ *ham-5^1-351^-gfp* (WD40 domain only) and Δ*ham-5*+ *ham-5* ^Δ67–348^ *-gfp* (W/O WD40 domain) and three slant tubes with the point mutation mutants *ham-5* ^S506A^ *-gfp*, *ham-5* ^S506E^ *-gfp* and *ham-5^RRK1128AAA^*-GFP (B) Western blot showing the protein sizes and levels of full length HAM-5-GFP (black arrows) and HAM-5-GFP^Δ67–348^ (red arrows) in conidia and germlings in WT and Δ*ham-5* strains. The right panel shows molecular weight marker sizes (kD) (C) A representative Western blot showing the protein sizes of MAK-2-mCherry (68 kD), MEK-2-mCherry (83 kD), NRC-1-mCherry (128 kD) and SOFT-mCherry (167 kD). A molecular marker is given at the right indicating marker sizes in kilodalton (kD). (D) A representative Western blot showing the protein sizes of HAM-5^1-351^-GFP (65 kD), free GFP (27 kD) and HAM-5-GFP (210 kD). Molecular weight markers are given at the right (kD). (E) A representative Western blot showing the protein sizes of phosphorylated MAK-1 (47 kD) and phosphorylated MAK-2 (41 kD). Molecular weight markers are given on the right (kD). (F) Western blots showing a specific interaction between HAM-5-GFP (210 kD) and phosphorylated MAK-2 (40.6 kD) in WT cells, but not between HAM-5^Δ67-348^-GFP (180 kD) and phosphorylated MAK-2. Top panel shows Western of protein samples using anti-P42/44 antibodies. The middle panel shows anti-GFP immunoprecipitated proteins probed with anti-GFP antibodies. Lower panels shows Western blot of anti-GFP immunoprecipitated proteins probed with anti-P42/44 antibodies. The strain Δ*mak-2*+ HAM-5-GFP was used as a negative control.
(TIF)

Figure S5 **Localization of HAM-5^1-351^*-*GFP (WD40 domain) and H1-dsRed in WT germlings and SO-GFP localization in wild-type and Δ*ham-5* germlings.** (A) Upper left panel is bright field image (scale bar  = 10 µM), upper right panel shows GFP fluorescence (HAM-5^1-351^-GFP) localization in WT germlings during chemotropic interactions; note localization to CAT tip and to nuclei (white arrows). Lower left panel shows H1-dsRED localization in germlings (four nuclei (arrow) and to vacuoles). Lower right panel shows co-localization of HAM-5^1-351^ *-*GFP and H1-dsRED to four nuclei (red arrow points to one nucleus). (B) In the WT germlings that are not communicating or (C) in the Δ*ham-5* strain, SO-GFP shows cytoplasmic localization and is absent from the nucleus (black areas). Size bar  = 10 µM.
(TIF)

Figure S6 **HAM-5-GFP and MAK-2-mCherry oscillate to opposite sites of fusion in hyphae.** (A) HAM-5-GFP and MAK-2-mCherry oscillate together every four minutes to the tip of an homing hypha (T4) and receptive hypha (T8) (white arrows) at a site near a septum (red arrow). MAK-2-mCherry also localizes to nuclei (white arrow, lower right picture). (B) HAM-5-GFP localizes to puncta in fusing hyphae when HAM-5-GFP is concentrated at the tip or at sites surrounding the septum (T = 4 and T = 8). Once the hyphae have merged and cytoplasmic flow is observed (see bright field picture at T = 22 min), HAM-5-GFP puncta appearance and disappearance is coincident in both hyphae (T = 46 and T = 59). For graphical representation, see Figure 7C and for Movies S5. (C) Bright field image showing hyphal fusion at T = 22 minutes.
(TIF)

Figure S7 **Oscillatory appearance and disappearance of HAM-5-GFP to puncta is restricted to hyphal compartments surrounding the site of fusion.** A) Bright field image of a fusing hyphal pair in which septa (*) and site of fusion (red circle) are visible. Scale bar  = 10 µm. B) Ten different septated hyphal compartments surrounding the site of fusion were assigned. (C) Appearance of HAM-5-GFP (maximal fluorescence intensity) and disappearance (minimal fluorescence intensity) was followed over time for each assigned fragment (Movie S5). (D) The fluorescence intensities for each assigned fragment from 66 frames (11 minutes) were plotted and a trend line was drawn through the data points. A full line is drawn through all graphs at places where fluorescence is low for fragments 2-9 and a dotted line when fluorescence was high in order to compare each graph for similar rhythmicity. *y* axis shows the ratio of relative fluorescence intensity (R.F.I.) in each fragment as compared to background. *x* axis shows time. (E) Fragments showing comparable graphs in (D) are color coded similarly. Fragments 2 – 9 showed similar oscillation graphs and were coded blue as fragments 1 and 10 showed dissimilar oscillation patterns compared to the rest and were coded red.
(TIF)

Movie S1 **Oscillatory movement of HAM-5-GFP in two germlings undergoing chemotropic interactions.** A 90-minute movie compressed in 26 seconds of two *ham-5-gfp* germlings undergoing chemotropic interactions and cell fusion. During chemotropic interactions, oscillatory recruitment of HAM-5-GFP to the CAT tips and puncta of germlings and to the site of contact is observed. The left panel is composed of brightfield images, the right panel of fluorescent images.
(AVI)

Movie S2 **Oscillatory movement of HAM-5-GFP and MAK-2-mCherry to the same CAT tip in germlings undergoing chemotropic interactions.** A 30-minute movie compressed in 9 seconds of two *ham-5-gfp; mak-2-mCherry* germlings undergoing chemotropic interactions and cell fusion. Oscillatory recruitment of HAM-5-GFP and MAK-2-mCherry to the same CAT tip of germlings (co-oscillation of HAM-5 and MAK-2) and to the site of contact is observed. The upper left panel is made of brightfield images, the lower left of GFP-fluorescent images, the lower right panel of mCherry-fluorescent images and the upper right panel movie is of merged GFP and mCherry images.
(AVI)

Movie S3 **Oscillatory movement of HAM-5-GFP and SO-mCherry to opposite CAT tips in two germlings undergoing chemotropic interactions.** A 52-minute movie compressed in 15 seconds of two genetically identical *ham-5-gfp; so-mCherry* germlings undergoing chemotropic interactions and cell fusion. Oscillatory recruitment of HAM-5-GFP and SO-mCherry to opposite CAT tips of the germlings is observed. Ham-5-GFP is also observed at the site of germling contact, while SO-mCherry is not. After fusion and cytoplasm mixing, the HAM-5-FGP and SO-mCherry fluorescent proteins are observed in both germlings. The upper left panel is made of brightfield images, the lower left of GFP-fluorescent images, the lower right of mCherry-fluorescent images and the upper right panel is a movie of merged GFP and mCherry fluorescent images.
(AVI)

Movie S4 **MEK-2-mCherry localizes to puncta and to septa in wild type hyphae, but only localizes to septa in hyphae of a Δ*ham-5* strain.** Two 4.5-minute movies compressed into 1 second showing MEK-2-mCherry in WT (*mek-2-mCherry*; upper panels and in Δ*ham-5* (Δ*ham-5; mek-2-mCherry*; lower panels) hyphae. Septal localization of MEK-2-mCherry is observed in both strains, but localization of MEK-2-mCherry to puncta is only observed in WT hyphae. The left panels are made of brightfield images, the right panels of fluorescent images.
(AVI)

Movie S5 **Oscillatory movement of HAM-5-GFP and MAK-2-mCherry to punta, to the hyphal tip and eventual site of contact in hyphae undergoing chemotropic interactions and fusion.** A 60-minute movie compressed in 17 seconds of two hyphae undergoing chemotropic interactions and cell fusion. Oscillatory co-recruitment of HAM-5-GFP and MAK-2-mCherry to puncta, to hyphal tips undergoing chemotropic growth, to sites surrounding the septum, and to the site of contact is observed. MAK-2-mCherry is also observed in nuclei, but HAM-5-GFP is not. Alternating oscillation of both HAM-5-GFP and MAK-2-mCherry to puncta and the hyphal tip in the two homing hyphae is observed before fusion, but after fusion, the oscillation of HAM-5-GFP and MAK-2-mCherry to puncta and septa is observed simultaneously in both hyphae. The upper left panel is made of brightfields images, the lower left panel of GFP-fluorescent images, the lower right panel of mCherry-fluorescent images and the upper right panel is of merged GFP and mCherry fluorescent images.
(AVI)

Movie S6 **Oscillation of HAM-5-GFP to puncta is restricted to hyphal compartments surrounding the site of fusion.** An 11-minute movie compressed in 9 seconds of two hyphae that are undergoing fusion. Oscillatory recruitment of HAM-5-GFP to puncta throughout the hyphae, to septa and to the site of contact is observed. Co-oscillation of HAM-5-GFP to puncta is seen in hyphae that have completed fusion, but co-oscillation is restricted to a few hyphal compartment distal to the site of fusion and is not observed in compartments further away (See Figure S7). The left panel is made of brightfield images, the right of GFP fluorescent images.
(AVI)

Table S1 **Phosphopeptides that show lower abundance in the 1NM-PP1 treated cells compared to untreated cells.** List of genes, identified phosphorylation site and peptides, *N. crassa* locus (if characterized), predicted annotation (https://www.broadinstitute.org/annotation/genome/neurospora/MultiHome.html) and whether deletion mutants in identified genes are capable of undergoing germling fusion.
(DOCX)

Table S2 **Strains used in this study. Strain name, genotype and reference for strains generated or obtained for this study.**
(PDF)

Dataset S1 **Phosphopeptides identified in this study.** Page 1: Dataset S1 labeling summary. Page 2: All phosphopeptides identified in this study. Page 3: List of all unique proteins identified from phosphopeptide data. Page 4: Data for biological repeats 1 and 2 (experiment 1). Page 5: Data for biological repeats 3 and 4 (experiment 2). Page 6: 455 phosphopeptides with significant abundance change (p<0.05) in 1NM-PP1 treated cells (*mak-2^Q100G^*) relative to controls. Page 7: Phosphopeptides showing an increase in abundance (p<0.05) of at least 1.5 fold. Page 8: Functional category analyses [30] of genes encoding phosphopeptides that increased in abundance after treatment with 1NM-PP1. Page 9: Phosphopeptides that showed significant (p<0.05) decreased abundance in 1NM-PP1 treated *mak-2^Q100G^* cells relative to controls. Page 10: Functional category analysis [30] of genes encoding phosphopeptides that showed a decrease in abundance.
(XLS)
