# Supplementary figures and images for: HAM-5 Functions As a MAP Kinase Scaffold during Cell Fusion in Neurospora crassa
Source: PLoS Genet. 2014 Nov 20;10(11):e1004783. doi: 10.1371/journal.pgen.1004783 (PMC4238974; doi:10.1371/journal.pgen.1004783)

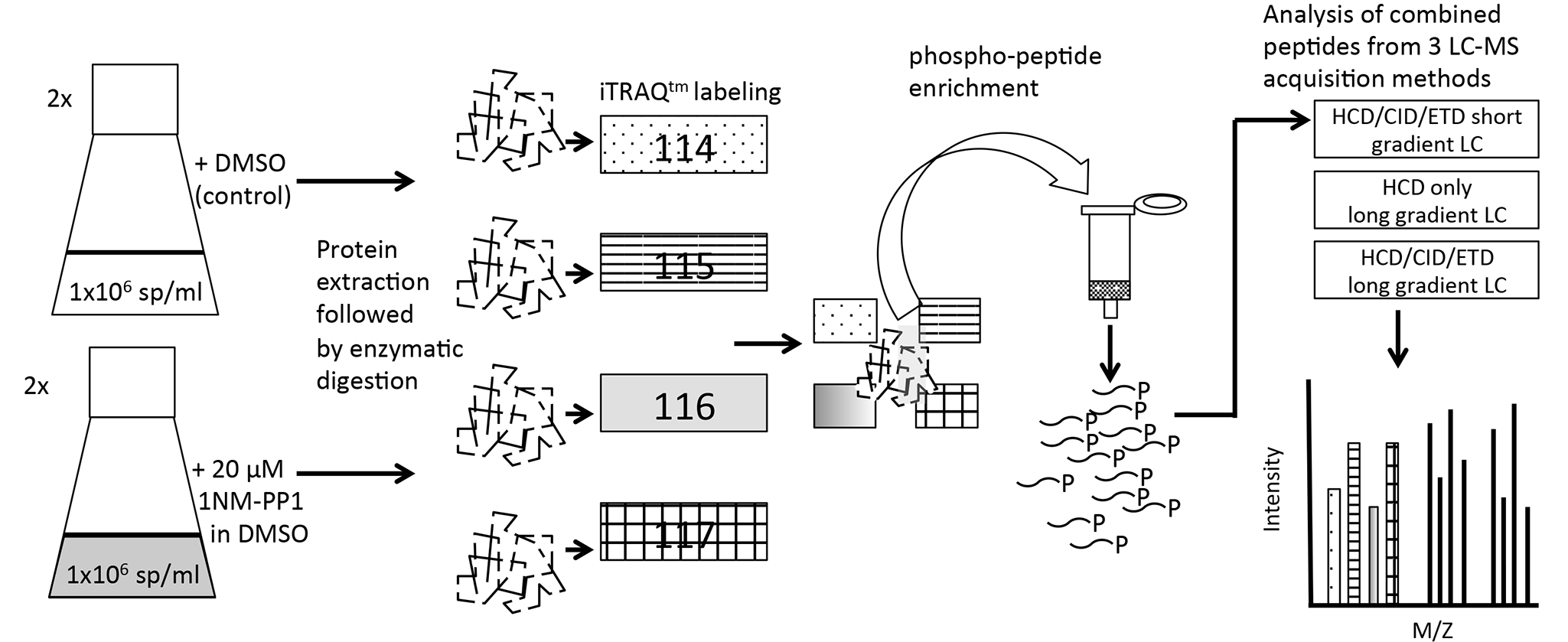

Supplement: Figure S1 — Schematic overview of protein samples of DMSO (control) and 10 µM 1NM-PP1 in DSMO treated cells (two replicas each), downstream processing and mass spectrometry analysis. Two flasks of control and 1NM-PP1 treated mak-2Q100G 5-hr old germlings were used to collect protein (left). Each of the four protein samples was digested with trypsin and subsequently treated with a barcoded iTRAQ label. The barcoded samples were mixed and subjected to immobilized metal affinity chromatography to enrich for phosphopeptides. The phosphopeptides were identified using liquid chromatography-mass spectrometry (LC-MS) with three different LC-MS acquisition methods. Statistical analyses were performed to identify peptides that differed in abundance between treatments. (TIF) [file pgen.1004783.s001.tif]

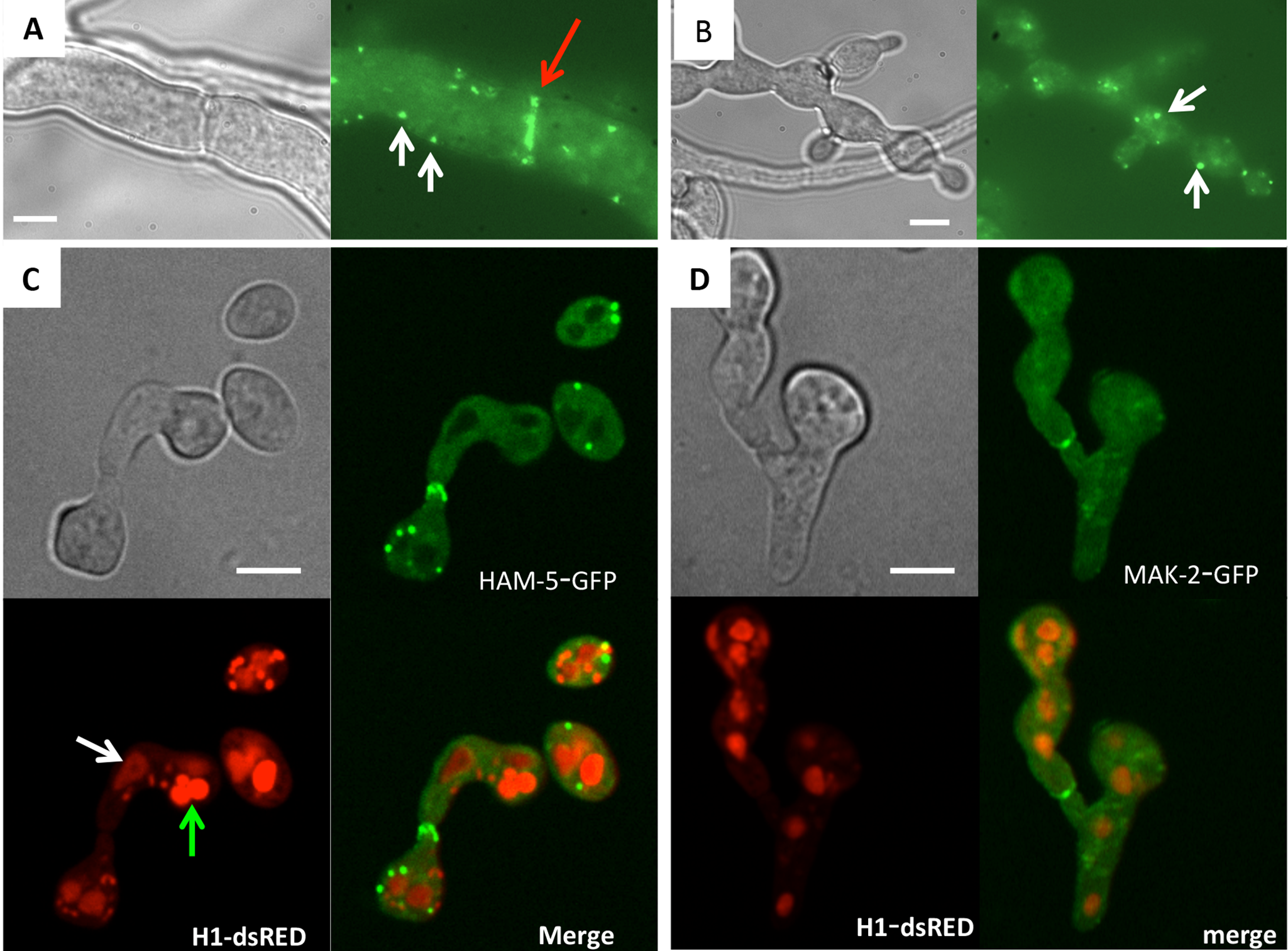

Supplement: Figure S2 — HAM-5-GFP localizes to puncta in conidia and mature hyphae, but is excluded from the nucleus. (A) Localization of HAM-5-GFP to puncta in a hypha (white arrows) and to the septum (red arrow). The left panel shows a bright field image (scale bar = 10 µM). (B) Localization of HAM-5-GFP to puncta in conidia (white arrows). The panel on the left shows a bright field image (scale bar = 10 µM). (C) Composite of HAM-5-GFP and H1-dsRED during germling fusion. Upper left panel is a bright field image (scale bar = 10 µM), upper right panel shows HAM-5-GFP fluorescence, lower left panel shows H1-dsRED fluorescence and lower right panel shows the composite of HAM-5-GFP and H1-dsRED fluorescent images. H1-dsRED localizes to the nucleus and is visible in vacuolar structures (lower left, white arrow and green arrows, respectively). (D) Composite of MAK-2-GFP and H1-dsRED during germling fusion. Upper left panels is a bright field image (scale bar = 10 µM), upper right panel shows MAK-2-GFP fluorescence, lower left panel shows H1-dsRED fluorescence and the lower right panel shows the composite of MAK-2-GFP and H1-dsRED images. (TIF) [file pgen.1004783.s002.tif]

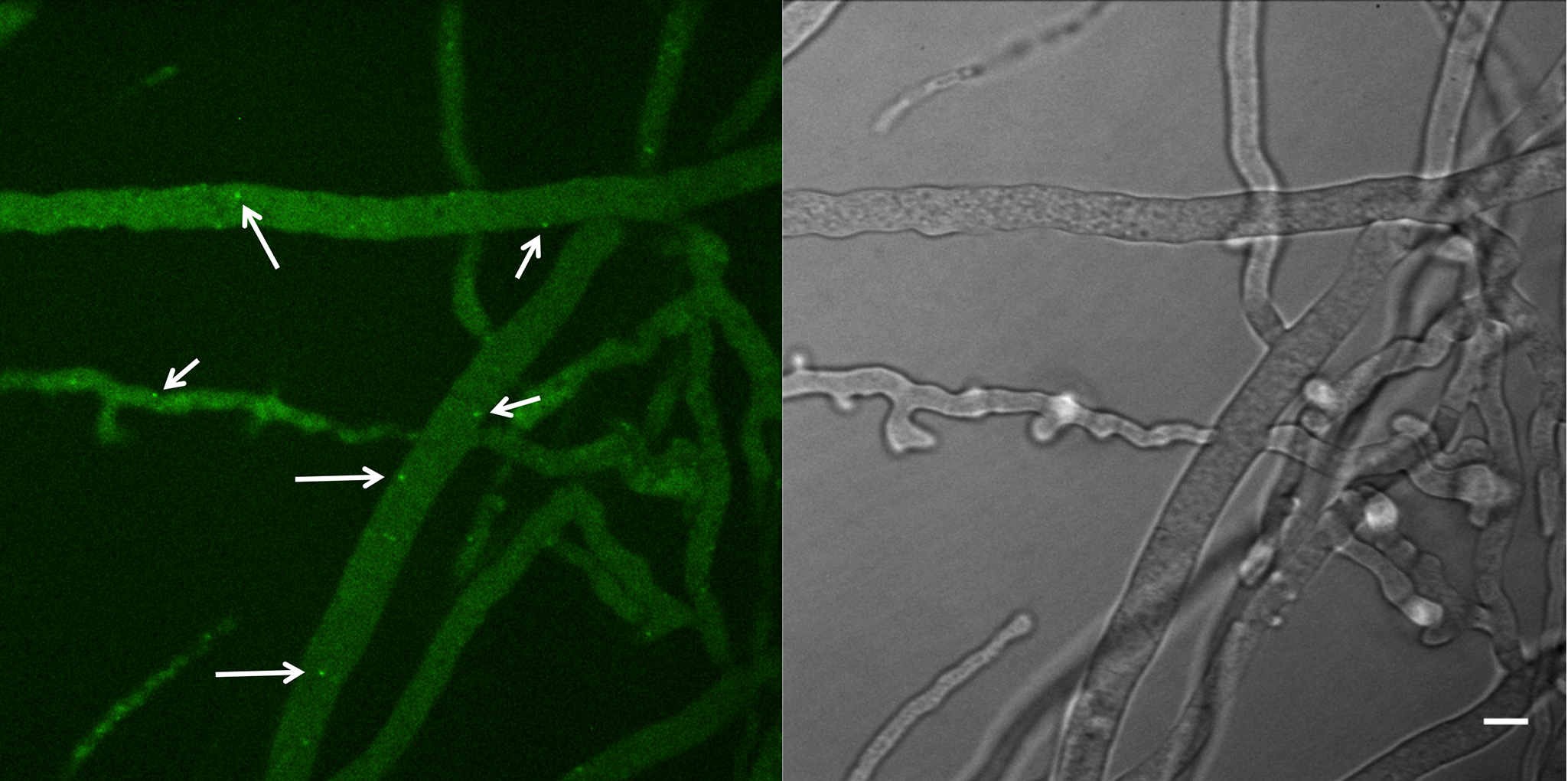

Supplement: Figure S3 — Localization of HAM-5-GFP in hyphae when driven by its native promoter. Left panel: GFP fluorescence images showing HAM-5-GFP localization to puncta in hyphae (arrows). The right panel is a bright field image. Scale bar = 10 µM. (TIF) [file pgen.1004783.s003.tif]

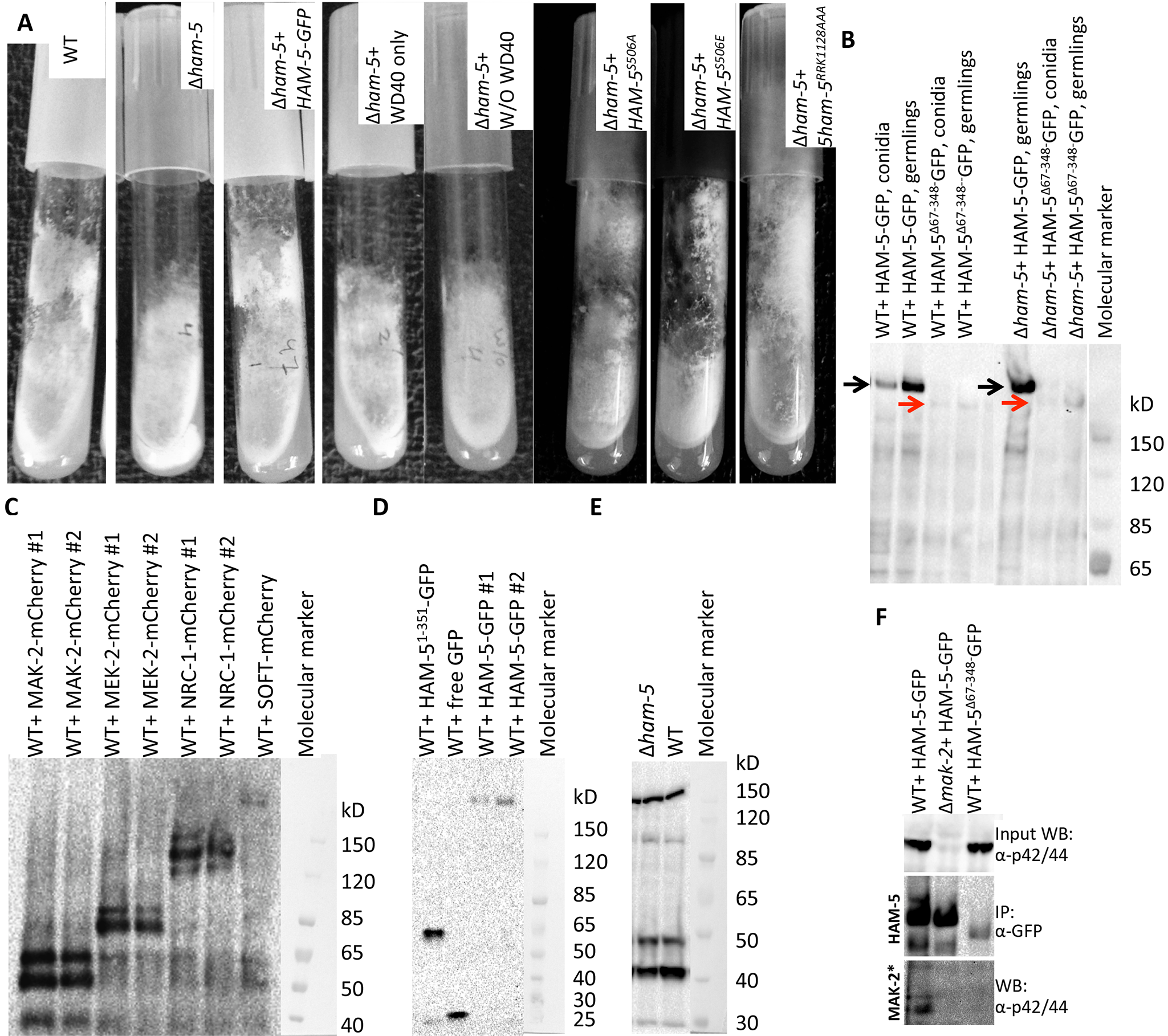

Supplement: Figure S4 — The WD40 domain is required for HAM-5 stability and function, but does not complement the Δham-5 growth defect. (A) Slant tubes with WT, Δham-5, Δham-5+ ham-5-gfp, Δham-5+ ham-51-351-gfp (WD40 domain only) and Δham-5+ ham-5 Δ67–348 -gfp (W/O WD40 domain) and three slant tubes with the point mutation mutants ham-5 S506A -gfp, ham-5 S506E -gfp and ham-5RRK1128AAA-GFP (B) Western blot showing the protein sizes and levels of full length HAM-5-GFP (black arrows) and HAM-5-GFPΔ67–348 (red arrows) in conidia and germlings in WT and Δham-5 strains. The right panel shows molecular weight marker sizes (kD) (C) A representative Western blot showing the protein sizes of MAK-2-mCherry (68 kD), MEK-2-mCherry (83 kD), NRC-1-mCherry (128 kD) and SOFT-mCherry (167 kD). A molecular marker is given at the right indicating marker sizes in kilodalton (kD). (D) A representative Western blot showing the protein sizes of HAM-51-351-GFP (65 kD), free GFP (27 kD) and HAM-5-GFP (210 kD). Molecular weight markers are given at the right (kD). (E) A representative Western blot showing the protein sizes of phosphorylated MAK-1 (47 kD) and phosphorylated MAK-2 (41 kD). Molecular weight markers are given on the right (kD). (F) Western blots showing a specific interaction between HAM-5-GFP (210 kD) and phosphorylated MAK-2 (40.6 kD) in WT cells, but not between HAM-5Δ67-348-GFP (180 kD) and phosphorylated MAK-2. Top panel shows Western of protein samples using anti-P42/44 antibodies. The middle panel shows anti-GFP immunoprecipitated proteins probed with anti-GFP antibodies. Lower panels shows Western blot of anti-GFP immunoprecipitated proteins probed with anti-P42/44 antibodies. The strain Δmak-2+ HAM-5-GFP was used as a negative control. (TIF) [file pgen.1004783.s004.tif]

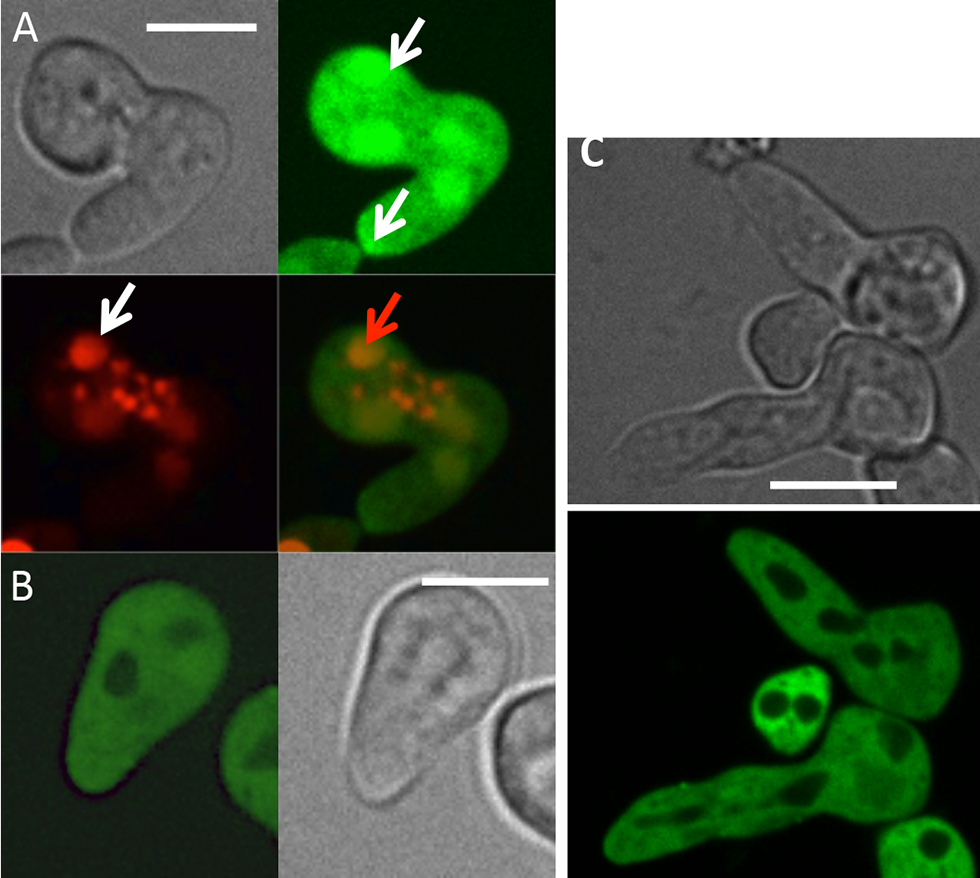

Supplement: Figure S5 — Localization of HAM-51-351-GFP (WD40 domain) and H1-dsRed in WT germlings and SO-GFP localization in wild-type and Δham-5 germlings. (A) Upper left panel is bright field image (scale bar = 10 µM), upper right panel shows GFP fluorescence (HAM-51-351-GFP) localization in WT germlings during chemotropic interactions; note localization to CAT tip and to nuclei (white arrows). Lower left panel shows H1-dsRED localization in germlings (four nuclei (arrow) and to vacuoles). Lower right panel shows co-localization of HAM-51-351 -GFP and H1-dsRED to four nuclei (red arrow points to one nucleus). (B) In the WT germlings that are not communicating or (C) in the Δham-5 strain, SO-GFP shows cytoplasmic localization and is absent from the nucleus (black areas). Size bar = 10 µM. (TIF) [file pgen.1004783.s005.tif]

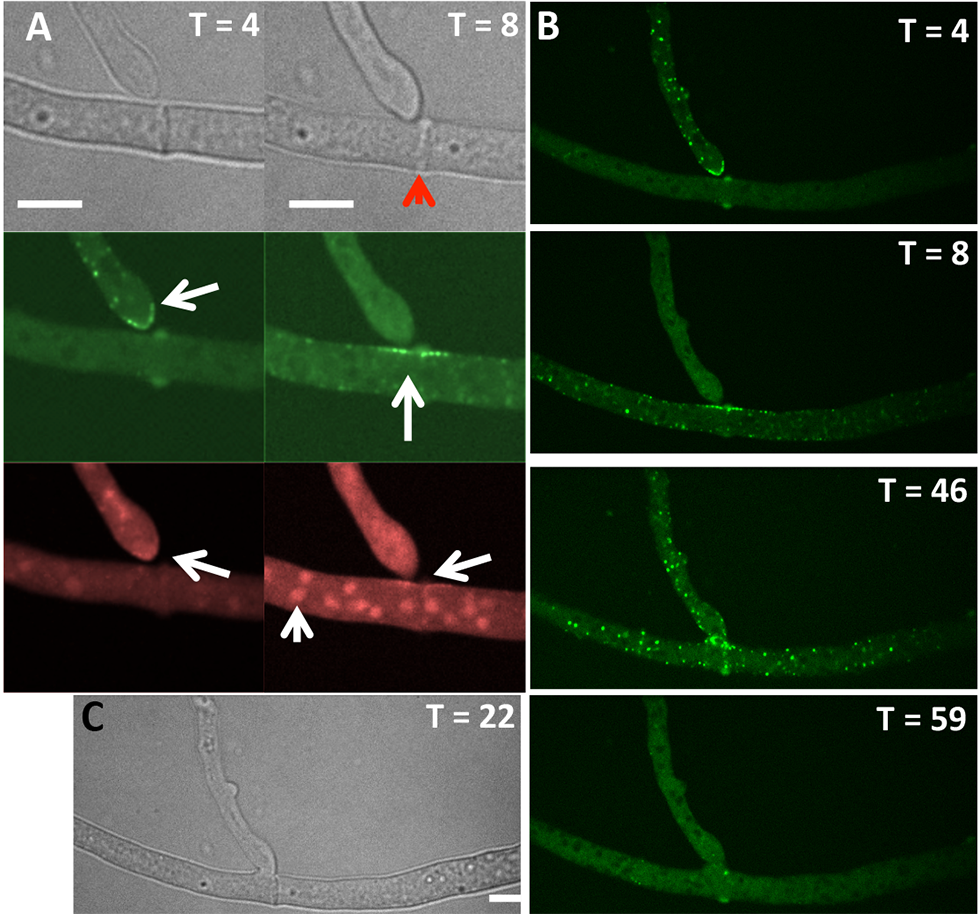

Supplement: Figure S6 — HAM-5-GFP and MAK-2-mCherry oscillate to opposite sites of fusion in hyphae. (A) HAM-5-GFP and MAK-2-mCherry oscillate together every four minutes to the tip of an homing hypha (T4) and receptive hypha (T8) (white arrows) at a site near a septum (red arrow). MAK-2-mCherry also localizes to nuclei (white arrow, lower right picture). (B) HAM-5-GFP localizes to puncta in fusing hyphae when HAM-5-GFP is concentrated at the tip or at sites surrounding the septum (T = 4 and T = 8). Once the hyphae have merged and cytoplasmic flow is observed (see bright field picture at T = 22 min), HAM-5-GFP puncta appearance and disappearance is coincident in both hyphae (T = 46 and T = 59). For graphical representation, see Figure 7C and for Movies S5. (C) Bright field image showing hyphal fusion at T = 22 minutes. (TIF) [file pgen.1004783.s006.tif]

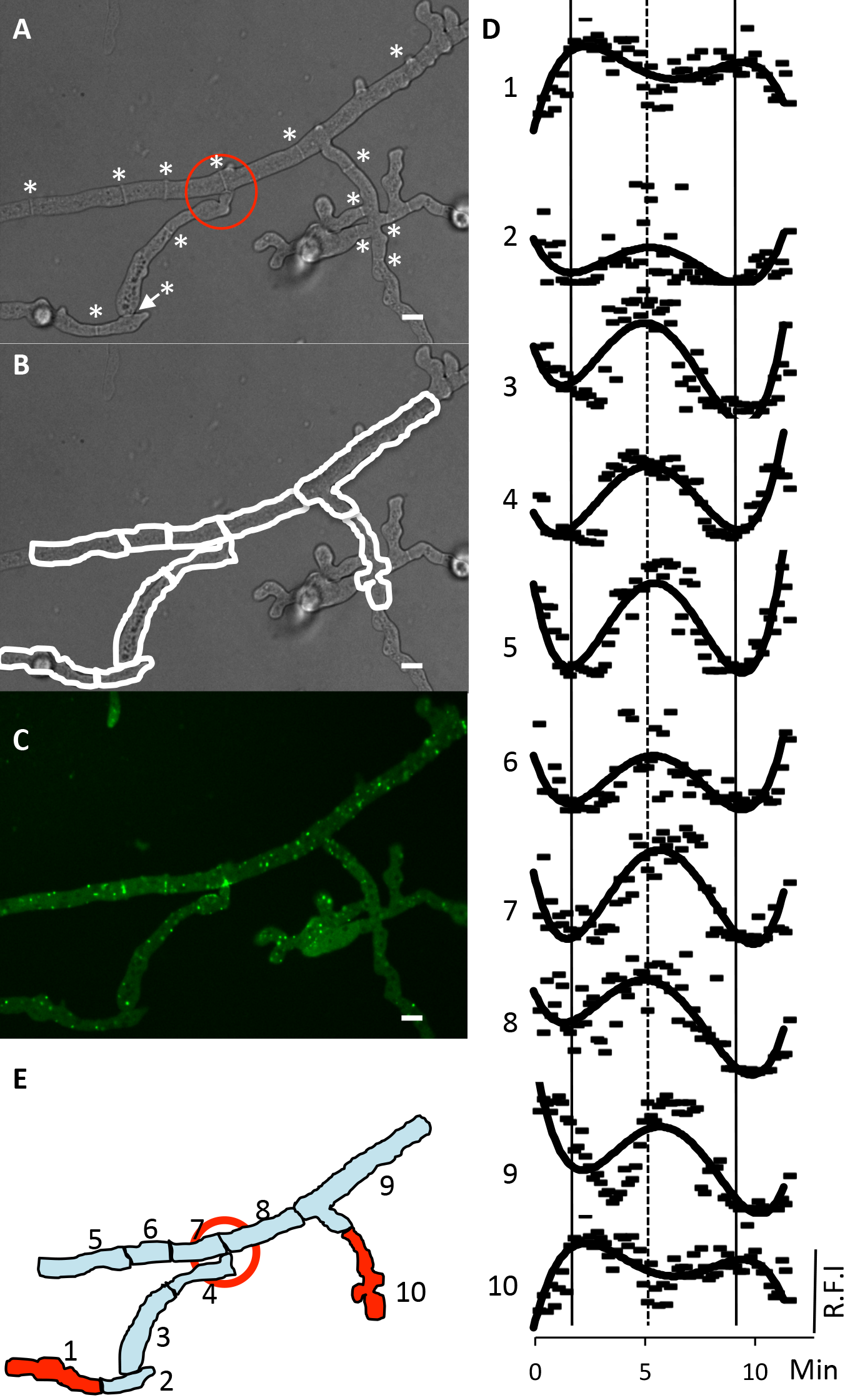

Supplement: Figure S7 — Oscillatory appearance and disappearance of HAM-5-GFP to puncta is restricted to hyphal compartments surrounding the site of fusion. A) Bright field image of a fusing hyphal pair in which septa (*) and site of fusion (red circle) are visible. Scale bar = 10 µm. B) Ten different septated hyphal compartments surrounding the site of fusion were assigned. (C) Appearance of HAM-5-GFP (maximal fluorescence intensity) and disappearance (minimal fluorescence intensity) was followed over time for each assigned fragment (Movie S5). (D) The fluorescence intensities for each assigned fragment from 66 frames (11 minutes) were plotted and a trend line was drawn through the data points. A full line is drawn through all graphs at places where fluorescence is low for fragments 2-9 and a dotted line when fluorescence was high in order to compare each graph for similar rhythmicity. y axis shows the ratio of relative fluorescence intensity (R.F.I.) in each fragment as compared to background. x axis shows time. (E) Fragments showing comparable graphs in (D) are color coded similarly. Fragments 2 – 9 showed similar oscillation graphs and were coded blue as fragments 1 and 10 showed dissimilar oscillation patterns compared to the rest and were coded red. (TIF) [file pgen.1004783.s007.tif]
