# Supplementary material for: HAM-5 Functions As a MAP Kinase Scaffold during Cell Fusion in Neurospora crassa
Source: PLoS Genet. 2014 Nov 20;10(11):e1004783. doi: 10.1371/journal.pgen.1004783 (PMC4238974; doi:10.1371/journal.pgen.1004783)
Supplement: Table S1 — Phosphopeptides that show lower abundance in the 1NM-PP1 treated cells compared to untreated cells. List of genes, identified phosphorylation site and peptides, N. crassa locus (if characterized), predicted annotation (https://www.broadinstitute.org/annotation/genome/neurospora/MultiHome.html) and whether deletion mutants in identified genes are capable of undergoing germling fusion. (DOCX) [file pgen.1004783.s014.docx]

**Table S1: Phosphopeptides that decreased in abundance after treatment with 1NM-PP1**

| **NCU #** | **Phosphorylated peptide** | **locus** | **Annotation** | **Germling fusion** |
| --- | --- | --- | --- | --- |
| NCU00420 | VIQS*APLGQSFPGR |  | hypothetical protein | yes |
| NCU00424 | SDRDDWS*PGPAVPESERR |  | Spo76 protein | yes |
| NCU00457 | T*VSEAPEAGSTASTDSK |  | translation initiation factor 4B | yes |
| NCU00521 | SPVS*PTAGEFTFAPRQS*LDSAR |  | hypothetical protein | yes |
| NCU00575 | MTMS*SPEGFKDENVFR |  | glucokinase | yes |
| NCU00575 | MT*MSSPEGFKDENVFR |  | glucokinase | yes |
| NCU00575 | RMT*MSSPEGFKDENVFR |  | glucokinase | yes |
| NCU00627 | RRS*PS*PVHMHER |  | hypothetical protein | yes |
| NCU00725 | SSAGIWGTPAPS*TPTSGSRPTTGGNHGLDDLLG |  | epsin-3 | yes |
| NCU00935 | RT*SLFGNLSFGK |  | hypothetical protein | yes |
| NCU00935 | T*SLFGNLSFGK |  | hypothetical protein | yes |
| NCU00935 | RTS*LFGNLSFGKK |  | hypothetical protein | yes |
| NCU00935 | RTSLFGNLS*FGK |  | hypothetical protein | yes |
| NCU00935 | RT*SLFGNLSFGKK |  | hypothetical protein | yes |
| NCU00935 | RTS*LFGNLS*FGK |  | hypothetical protein | yes |
| NCU00935 | RTS*LFGNLSFGK |  | hypothetical protein | yes |
| NCU00980 | QRS*IT*PSAPAGAK |  | hypothetical protein | n/d |
| NCU01166 | LVEEDENDTITS*PTTPHFGVK |  | microcycle blastoconidiation | n/d |
| NCU01240 | WEVHGILT*SAILS*DGLLY*HR |  | hypothetical protein | yes |
| NCU01345 | R.SGPLS*PAMLSGPTTSDYFGDHIR | *asl-1* | *ascospore lethal-1* | yes |
| NCU01728 | SLNDLDGGIGGFGFTGQHNPIAYNSPYSQSIPSTAPGS*PR |  | 6-phosphofructo-2-kinase | n/d |
| NCU01789 | HEVPRS*PDDAKVVDLFK | *ham-5* | *hyphal anastomosis-5* | no |
| NCU02004 | VQRPSLS*S*SMRSSSYLQEHQQYR |  | phosphoserine phosphatase | n/d |
| NCU02036 | ES*TPSGIKR |  | tRNA-splicing endonuclease | yes |
| NCU02078 | S*T*ASPVPMFKPGR |  | hypothetical protein | yes |
| NCU02078 | S*TAS*PVPMFKPGR |  | hypothetical protein | yes |
| NCU02209 | TQT*EATSDSYPGTADASPFDSPLER |  | delta-12 fatty acid desaturase | yes |
| NCU02505 | ANS*TIMQLNK | *suc* | succinate | n/d |
| NCU02505 | ANST*IMQLNK | *suc* | succinate | n/d |
| NCU02505 | IRANS*TIMQLNK | *suc* | succinate | n/d |
| NCU02696 | ASS*PVRLGDVK |  | pre-mRNA-processing ATP-dependent RNA helicase prp-5 | n/d |
| NCU02720 | KLS*VSGNNEQAR |  | hypothetical protein | yes |
| NCU02828 | AKT*PGLGNK |  | condensin complex component cnd2 | n/d |
| NCU02830 | APS*FDESPDRLLQMLR |  | ARF GTPase activator | n/a |
| NCU03070 | SLALNSGGPRS*PFPIDR |  | hypothetical protein | yes |
| NCU03253 | NNAPGEGTT*SPGPR |  | hypothetical protein | n/a |
| NCU03500 | KFS*INR |  | aminotransferase | yes |
| NCU03725 | KHS*RT*QT*PSQPR | *vib-1* | vegetative incompatibility blocked-1 | yes |
| NCU03990 | AIDS*PERPAHDDLDVAE |  | hypothetical protein | n/a |
| NCU04164 | T*PTPGKYFGPPK |  | hypothetical protein | yes |
| NCU04314 | RHSNVSNPS*VPHY*LEQFK |  | hypothetical protein | yes |
| NCU04363 | SNT*PLPGRPK |  | hypothetical protein | n/d* |
| NCU04732 | GASFLNFLKS*PK | *ham-11* | hyphal anastomosis-11 | No |
| NCU04732 | GAS*FLNFLKSPK | *ham-11* | hyphal anastomosis-11 | No |
| NCU04807 | SLS*DAGIKPVEFGPR |  | universal stress protein family domain-containing protein | n/a |
| NCU04924 | NS*FGASLPIPR | *cut-1* | similar to phosphatidyl synthase | yes |
| NCU04924 | LSS*VTFPGGVAEALQEASGGTLPLKPTR | *cut-1* | similar to phosphatidyl synthase | yes |
| NCU04924 | LSSVT*FPGGVAEALQEASGGTLPLKPTR | *cut-1* | similar to phosphatidyl synthase | yes |
| NCU04924 | LSRNS*FGASLPIPR | *cut-1* | similar to phosphatidyl synthase | yes |
| NCU04924 | RLS*RNS*FGASLPIPR | *cut-1* | similar to phosphatidyl synthase | yes |
| NCU04924 | RLS*RNSFGASLPIPR | *cut-1* | similar to phosphatidyl synthase | yes |
| NCU04924 | SPDAHADLYMHNLS*ASPSLKER | *cut-1* | similar to phosphatidyl synthase | yes |
| NCU04924 | LSRNS*FGASLPIPR | *cut-1* | similar to phosphatidyl synthase | yes |
| NCU05041 | IDEHDIARS*PGTVGLEETGSVDR |  | trehalose-phosphatase | yes |
| NCU05041 | TESSLPGHLRPSVINVPVT*PGISR |  | trehalose-phosphatase | yes |
| NCU05041 | TESSLPGHLRPS*VINVPVTPGISR |  | trehalose-phosphatase | yes |
| NCU05289 | NAT*EDPDAMEVDTPK |  | nucleolar GTP-binding protein 1 | n/d* |
| NCU05364 | SNSQSYFTHKPT*GTPLAS*PR |  | tyrosine-protein phosphatase non-receptor type 6 | yes |
| NCU06247 | TAAS*NETTSREAT*PR |  | hypothetical protein | yes |
| NCU06247 | LEVPHS*PR |  | hypothetical protein | yes |
| NCU06249 | LMDVMATPFLAVDSGPQDHLPS*PAR |  | serine/threonine protein kinase | n/d |
| NCU06338 | KVADDAFDMEDS*E |  | DNA topoisomerase 2 | n/d* |
| NCU06644 | AS*TVGSPDEAQPAK |  | hypothetical protein | n/d* |
| NCU06644 | GTT*AELLGAVPVNTELFK |  | hypothetical protein | n/d* |
| NCU07002 | TSS*PDRQPPTGPSSSR |  | related to regulatory protein RLR1 | n/d* |
| NCU07010 | S*FLHGFRPGPGPESELPER |  | conserved hypothetical protein | yes |
| NCU07024 | IQDPQMT*GY*VSTR | *os-2* | osmotic sensitive-2 | yes |
| NCU07389 | INDVIS*PLESVSIVGIEQLMPKPHSCSK | *ham-9* | hyphal anastamosis-9 | no |
| NCU07400 | DAGS*GSLPRTPTDLVPPGPALK |  | hypothetical protein | yes |
| NCU07551 | LLPLELLES*DDEDDEVPRR |  | hypothetical protein | yes |
| NCU07685 | KS*FGTEGAER |  | hypothetical protein | yes |
| NCU07690 | NKS*YITR |  | methylenetetrahydrofolate reductase 1 | yes |
| NCU07868 | TPSSTATPDS*PR |  | hypothetical protein | yes |
| NCU07868 | SVEAPQPSAALQSLRS |  | hypothetical protein | yes |
| NCU07868 | SVEAPQPS*AALQSLR |  | hypothetical protein | yes |
| NCU07868 | TST*PVSTPK |  | hypothetical protein | yes |
| NCU08330 | KGS*MSFK |  | hypothetical protein | yes |
| NCU08362 | S*LSATPAQSASK |  | hypothetical protein | yes |
| NCU08362 | SLS*AT*PAQSASK |  | hypothetical protein | yes |
| NCU08377 | QTT*APGFTTK | *cr-1* | crisp-1 | yes |
| NCU08377 | QT*TAPGFTTK | *cr-1* | crisp-1 | yes |
| NCU08423 | AKVS*PVADQASAK |  | hypothetical protein | n/d* |
| NCU08423 | AASAQTAET*TTSHK |  | hypothetical protein | n/d* |
| NCU08811 | KPTPPIS*R |  | arf GTPase-activating protein | n/d |
| NCU08859 | RSS*S*VAGSRPGLSGSR |  | hypothetical protein | yes |
| NCU09068 | TAS*T*PGLSAR | *nit-2* | nitrate nonutilizer-2 | yes |
| NCU09118 | NT*DMDVDSDDDEPLGK |  | DNA topoisomerase I | n/d* |
| NCU09118 | T*QHKQPDS*DSDDEPIAK |  | DNA topoisomerase I | n/d* |
| NCU09130 | ILTLS*PR |  | hypothetical protein | n/d |
| NCU09195 | HRES*TLDPLTR | *gpr-6* | G-protein-coupled receptor-6 | yes |
| NCU09212 | Y*DFRSPGAVNLR | *camk-4* | calcium/calmodulin-dependent kinase-4 | yes |
| NCU09352 | DSGRGGS*AAPPPLKEGEDSLQIMVPDR |  | far upstream element-binding protein 2 | yes |
| NCU09352 | GGSAAPPPLKEGEDS*LQIMVPDR |  | far upstream element-binding protein 2 | yes |
| NCU09609 | KS*LTPAPR |  | hypothetical protein | yes |
| NCU09657 | GFPTVNTLGGWPTAPSVGT*PDRER |  | hypothetical protein | yes |
| NCU09842 | GFS*VDPEENAGYMTEYVATR | *mak-1* | MAP kinase | no |
| NCU11427 | S*ADYDRYDADPAILSDDFAGMK |  | CUE domain-containing protein | n/a |
| NCU17271 | HVSRAVELAMS*K |  | hypothetical protein | n/a |

n/a = not available, deletion strain is not available from the deletion database (69).

n/d = not determined, deletion is present as heterokaryon in the deletion database

n/d* = not determined, deletion is present as heterokaryon in the deletion database but ascospore lethality that co-segregated with the hygromycin marker was observed. Deletion might be (ascospore) lethal.
