# Supplementary material for: HAM-5 Functions As a MAP Kinase Scaffold during Cell Fusion in Neurospora crassa
Source: PLoS Genet. 2014 Nov 20;10(11):e1004783. doi: 10.1371/journal.pgen.1004783 (PMC4238974; doi:10.1371/journal.pgen.1004783)
Supplement: Table S2 — Strains used in this study. Strain name, genotype and reference for strains generated or obtained for this study. (PDF) [file pgen.1004783.s015.pdf]

Table S2: Strains used in the study

**Name**	**Genotype**	**Reference**[1]
FGSC6103	*his-3 A*	FGSC
MAL-1	*his-3::Pccg-1-mak-2^{Q100G}; Δmak-2 A*	[1]
FGSC988	Oak Ridge WT *a*	FGSC
FGSC2489	Oak Ridge WT *A*	FGSC
	his-3::P*ccg-1-gfp A*	This study
	his-3::P*ccg-1-mCherry A*	This study
AF-SoT8	*his-3*::P*ccg-1-so-gfp A*	[1]
AF-M512	*his-3::Pccg-1 mak-2-gfp; Δmak-2 A*	[1]
	his-3 Δham-5 a	This study
	his-3::Pccg-1-so-gfp A, Δham-5	This study
	his-3::P*ccg-mak-2-gfp A*, *Δham-5*	This study
	Δham-11 A	FGSC
	his-3 Δham-11 a	[2]
	Δham-7 A	FGSC
	his-3 Δham-7 a	[2]
AF-M621	*his-3*:: *Δmak-2* A	[1]
MAL-1	*his-3::Pccg-1-mak-2^{Q100G}; Δmak-2 A*	[1]
CR73–1	*his-3::Pccg-1-hH1-dsRED rid-1 A*	[3]
	his-3::Pccg-1-ham-5-gfp A	This study
	his-3::Ptef-1-ham-5-gfp A	This study
	his-3::Ptef-1-ham-5$^{1\text{-}351}$-gfp A	This study
	his-3::Ptef-1-ham-5$^{\Delta 67\text{-}348}$-gfp A	This study
	his-3::Ptef-1-mak-2-mCherry	This study
	his-3::Ptef-1-mek-2-mCherry	This study
	his-3::Ptef-1-nrc-1-mCherry	This study
	his-3::Ptef-1-so-mCherry	This study
	his-3::Ptef-1-mak-2-mCherry-Ptef-1-ham-5-gfp A	This study
	his-3::Ptef-1-mak-2-mCherry-Ptef-1-ham-5-gfp Δham-7 A	This study
	his-3::Ptef-1-mak-2-mCherry-Ptef-1-ham-5-gfp Δham-11 A	This study
	his-3::Ptef-1- ham-5^{RRK1128AAA}-gfp A	This study
	his-3::Ptef-1- ham-5^{S506A}-gfp A Δham-5	This study
	his-3::Ptef-1- ham-5^{S506E}-gfp A	This study
	his-3::Ptef-1- ham-5^{RRK1128AAA}-gfp Δham-5 A	This study
	his-3::Ptef-1- ham-5^{S506A}-gfp Δham-5 A	This study
	his-3::Ptef-1- ham-5^{S506E}-gfp Δham-5 A	This study

[1]References

1. Fleissner A, Leeder AC, Roca MG, Read ND, Glass NL (2009) Oscillatory recruitment of signaling proteins to cell tips promotes coordinated behavior during cell fusion. Proc Nat Acad Sci USA 106: 19387-19392.

2. Leeder AC, Jonkers W, Li J, Glass NL (2013) Germination and early colony establishment in *Neurospora crassa* requires a MAP kinase regulatory network. Genetics 195: 883-898.
3. Rasmussen CG, Morgenstein RM, Peck S, Glass NL (2008) Lack of the GTPase RHO-4 in *Neurospora crassa* causes a reduction in numbers and aberrant stabilization of microtubules at hyphal tips. Fungal Genet Biol 45: 1027-1039.
